# Patients' perspectives on a new delivery model in primary care: A propensity score matched analysis of patient‐reported outcomes in a Dutch cohort study

**DOI:** 10.1111/jep.13426

**Published:** 2020-06-17

**Authors:** Esther H. A. van den Bogaart, Marieke D. Spreeuwenberg, Mariëlle E. A. L. Kroese, Sofie J. M. van Hoof, Niels Hameleers, Dirk Ruwaard

**Affiliations:** ^1^ Department of Health Services Research Care and Public Health Research Institute (CAPHRI) Faculty of Health Medicine and Life Sciences Maastricht University Maastricht The Netherlands; ^2^ Research Center for Technology in Care Zuyd University of Applied Sciences Heerlen The Netherlands

**Keywords:** health policy, health services research, patient‐centered care, public health

## Abstract

**Rationale, aims and objective:**

Primary Care Plus (PC+) focuses on the substitution of hospital‐based medical care to the primary care setting without moving hospital facilities. The aim of this study was to examine whether population health and experience of care in PC+ could be maintained. Therefore, health‐related quality of life (HRQoL) and experienced quality of care from a patient perspective were compared between patients referred to PC+ and to hospital‐based outpatient care (HBOC).

**Methods:**

This cohort study included patients from a Dutch region, visiting PC+ or HBOC between December 2014 and April 2018. With patient questionnaires (T0, T1 and T2), the HRQoL and experience of care were measured. One‐to‐two nearest neighbour calliper propensity score matching (PSM) was used to control for potential selection bias. Outcomes were compared using marginal linear models and Pearson chi‐square tests.

**Results:**

One thousand one hundred thirteen PC+ patients were matched to 606 HBOC patients with well‐balanced baseline characteristics (SMDs <0.1). Regarding HRQoL outcomes, no significant interaction terms between time and group were found (*P* > .05), indicating no difference in HRQoL development between the groups over time. Regarding experienced quality of care, no differences were found between PC+ and HBOC patients. Only travel time was significantly shorter in the HBOC group (*P* ≤ .001).

**Conclusion:**

Results show equal effects on HRQoL outcomes over time between the groups. Regarding experienced quality of care, only differences in travel time were found. Taken as a whole, population health and quality of care were maintained with PC+ and future research should focus more on cost‐related outcomes.

## 1 INTRODUCTION

In 1978, the Declaration of Alma Ata identified primary health care as the key to achieving the goal of delivering better health care for all.^1^ Forty years later, the Global Conference on Primary Health Care came with a renewed declaration, in which the importance of focusing on primary health care was emphasized again.^2^ This new declaration states that, a focus on primary health care is still critical due to growing possibilities of technology, an ageing population and an increasing number of people suffering from multimorbidity.^3^, ^4^, ^5^ These developments lead to rapidly increasing health care costs in developed countries.^6^ According to the OECD, public expenditure on health‐ and long‐term care will increase to 9% of Gross Domestic Product in 2030 and even to 14% by 2060 in OECD countries. Therefore, the future sustainability of health care systems is at stake. Governments are challenged to continue providing accessible, equitable and affordable health care of adequate quality. In order to do so, policymakers are forced to redesign health care delivery models.^4^

As primary care functions as the door to the whole health care system, strengthening primary care is an important policy instrument in redesigning health care.^5^ An example is to shift hospital‐based medical specialists to the primary care setting without moving the hospital facilities.^7^, ^8^, ^9^, ^10^ This shift is a form of substitution, defined as: “the continual regrouping of resources across and within care settings, to exploit the best and least costly solutions in the face of changing needs and demands.”^11^ In 2013, regional collaboration initiatives in the Netherlands, focusing on substitution, were established to achieve the Triple Aim by improving the experience of care and the health of the population, and reducing the per capita costs.^12^, ^13^ Primary Care Plus (PC+) is one of these initiatives.^14^, ^15^, ^16^

With the Triple Aim framework, Berwick et al^13^ encourages health care organizations to reduce the cost of care, while at the same time increase the health of the population and the quality of care. In a study by Quanjel et al^17^ a PC+ intervention for patients with cardiology‐related complaints was evaluated based on the principles of the Triple Aim. In this PC+ setting, cardiologists provided consultations in the presence of similar diagnostic tools as in the hospital. The results showed that besides cost reduction, the health of the population and the quality of care did not decrease compared to care as‐usual. However, the present study focusses on a PC+ intervention in which hospital facilities are not available and therefore, medical specialists are only able to use their own expertise and experience. This forces them to use a generalist approach to analyse a patient's medical complaint.^18^

This study aims to evaluate whether the PC+ initiative (without the availability of the hospital facilities), is also able to increase the health of the population and the quality of care. Therefore, the health‐related quality of life (HRQoL) and the experienced quality of care from the patient's perspective are compared between patients referred to PC+ and patients referred directly to hospital‐based outpatient care (HBOC).

## 2 METHODS

### 2.1 Study design

This cohort study compared patient‐reported HRQoL and the experienced quality of care between patients referred to PC+ and patients referred to HBOC using propensity score matching (PSM). The reporting of this study follows the Strengthening the Reporting of Observational Studies in Epidemiology (STROBE) guidelines.^19^

The study is approved by the Medical Research and Ethics Committee of the Maastricht University Medical Centre (METC 14‐4‐136). Informed consent was obtained from all individual participants included in the study.

### 2.2 Setting and intervention

In the Maastricht‐Heuvelland region, located in the southern Netherlands, the primary care organization Care in Development (in Dutch “Zorg in ontwikkeling”), Maastricht University Medical Centre + (Maastricht UMC+), health insurance company VGZ and patient representative foundation “Burgerkracht Limburg” collaborate. In 2014, these organizations developed the PC+ intervention to substitute hospital‐based specialized care with primary care whereby GPs remain responsible for the patient. With two PC+ centres operating according to the same method, GPs within the region are able to refer non‐acute and low‐complex patients to a medical specialist in a neutral primary care setting. Based on the PC+ patients' profiles (listing relevant medical complaints for PC+), GPs' clinical expertise and shared decision‐making, GPs decide whether to refer a patient to PC+. In PC+, the medical specialist examines and/or treats the patient during a maximum of two consultations. Following PC+, the medical specialist refers the patient back to the GP with treatment advice, or, if necessary, refers the patient to HBOC for further diagnosis and/or treatment. Involved specialists are employed in the Maastricht UMC+ and perform PC+ consultations on a regular basis (weekly or biweekly). Like the Maastricht UMC+, the PC+ centres are both located in the city of Maastricht.

Besides the assumed benefits of PC+ being more informal and located closer to patients' homes, patients are exempt from paying a mandatory deductible for a consultation. In the Netherlands, GP consultations are fully covered by health insurance but for consulting a medical specialist, a yearly mandatory deductible is levied (€360 in 2014 and €385 since 2016).^20^ This mandatory deductible is determined by the government.^21^ Patients have to pay this deductible themselves before the health insurance company pays for specialized medical care.

### 2.3 Study population

In 2016, The Maastricht‐Heuvelland region consisted of 55 GP practices caring for a population of about 170 000 people.^22^ Patients eligible for inclusion were adult patients (≥18 years) from the Maastricht‐Heuvelland region visiting PC+ or HBOC between December 2014 and April 2018, with a referral to one of the medical specialties present in PC+ during the study period: dermatology, gynaecology, otolaryngology, internal medicine (including gastroenterology), neurology, ophthalmology, orthopaedics, rheumatology and urology. This study is part of a larger study, which requires 1830 patients per group (3660 patients in total).^16^

### 2.4 Data collection

After referral to PC+ or HBOC by the GP, all eligible patients were recruited by the Transmural Interactive Patient Platform (TIPP) for participation. TIPP plans and registers referrals to medical specialists in either PC+ or HBOC. TIPP informed patients about the study, and if interested, patients' contact details were sent to the research team. The research team then sent an information letter, informed consent and the first questionnaire (T0) to the patient by post or email. Patients were asked to return the informed consent and the questionnaire before the first consultation with the medical specialist. After the first consultation, a second questionnaire was sent within 1 week (T1) and a third questionnaire after 3 months (T2). The inclusion of patients started in December 2014 and continued until April 2018.

### 2.5 Outcome measures

#### 2.5.1 Baseline characteristics

**FIGURE 1:**
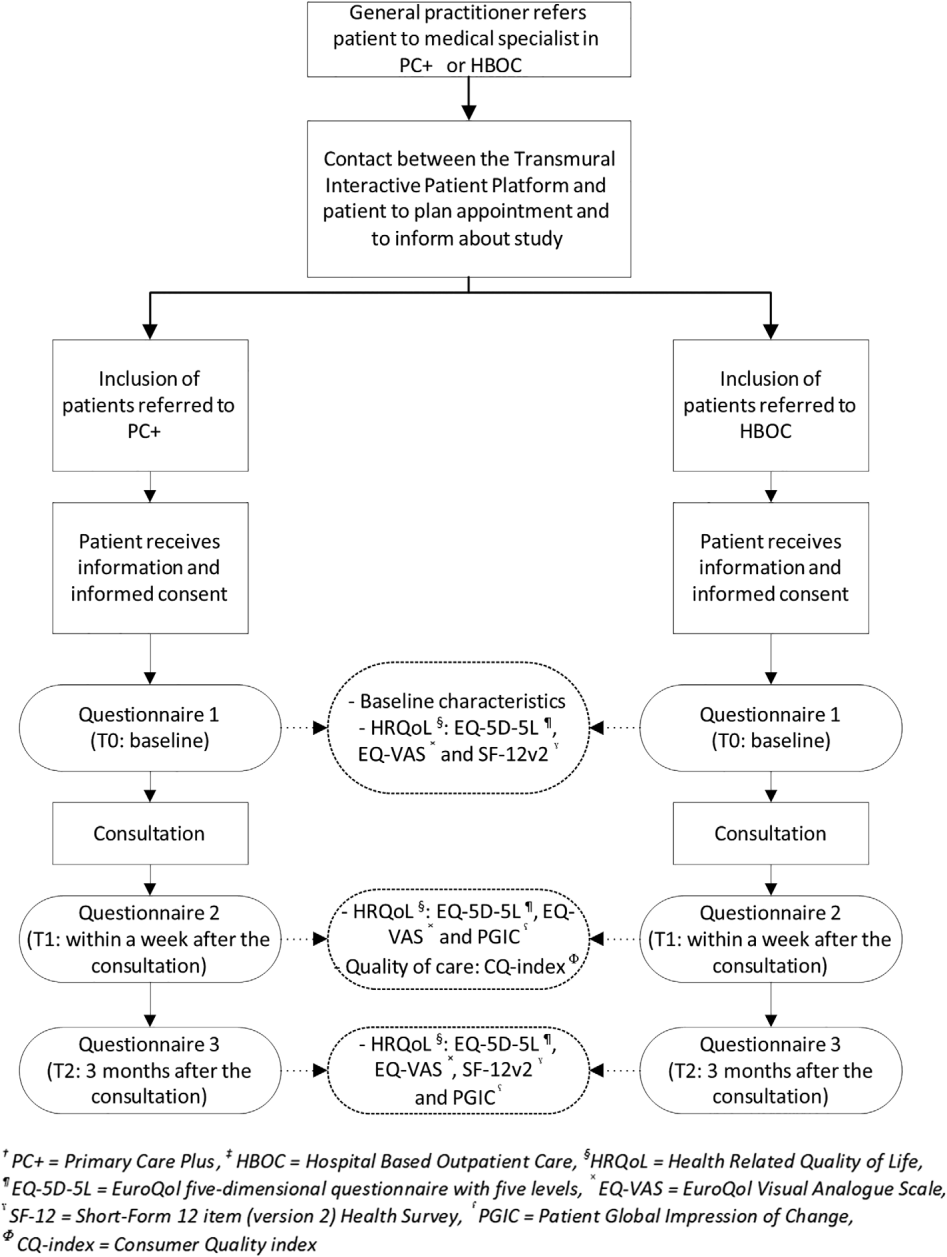
Flow chart of participating patients flow and questionnaire measurements

Baseline characteristics were collected during T0, including age in years, gender, native country and level of education (low vs medium vs high) (Figure 1). Collected risk factors included body mass index (BMI) calculated from reported height and weight, cigarette smoking (current vs former vs never) and alcohol use (yes vs no).

#### 2.5.2 Health‐related quality of life

To measure generic HRQoL, the EuroQol five‐dimensional questionnaire with five levels (EQ‐5D‐5L), including the EuroQol Visual Analogue Scale (EQ‐VAS) and the Short‐Form Health Survey version 2 (SF‐12v2) were used. Patients' perceptions of a change in their health status was evaluated with the Patient Global Impression of Change (PGIC) seven‐item response scale.

The EQ‐5D‐5L is a measure consisting of five questions on mobility, self‐care, pain/discomfort, usual activities and anxiety/depression with five response levels.^23^ A health state index score, ranging from −0.446 to 1 (worst to best imaginable health status), was calculated from individual health profiles using the Dutch utility tariff.^24^ The included EQ‐VAS is a 0 to 100 scale where respondents indicate their overall health. Both the EQ‐5D‐5L and the EQ‐VAS were measured at T0, T1 and T2. The minimal clinically important change in EQ‐5D‐5L is 0.04.^25^

The SF‐12v2 consists of 12 questions measuring the health status by means of two summary scores; a physical component summary (PCS) and a mental component summary (MCS).^26^ PCS and MCS scores range from 0 (lowest level of health) to 100 (highest level of health) and were obtained using the instrument developers' standard scoring algorithm.^26^ The SF‐12v2 was measured at T0 and T2. The minimal clinically important change for both PCS and MCS scores ranges between 3 and 5 points.^27^

With the PGIC scale, patients were able to indicate to what extent their health problem had changed after they consulted the medical specialist, ranging from 1 (very much improved) to 7 (very much worse).^28^ The PGIC scale was conducted at T1 and T2.

#### 2.5.3 Experienced quality of care

An influential and often used framework to measure quality of care is that of the Institute of Medicine, stating care must be safe, effective, patient‐centred, timely, efficient and equitable.^29^ Patient centeredness and timeliness are explicitly included in the Consumer Quality (CQ) index. The CQ‐index is a standardized method for measuring experiences of patients with health care.^30^ In this study, 21 items derived from the Dutch CQ‐index general practice^31^ and hospital outpatient care^32^ were used. Items can be divided into five domains: timeliness (3 items); treatment by the medical specialist (6 items); information provision and communication by the medical specialist (4 items); communication and collaboration between the medical specialist and GP (4 items); and the overall assessment of quality of care (4 items). Most item scores ranged from 1 to 4. However, travel time was measured in minutes on a continuous scale. Furthermore, the medical specialist and the outpatient clinic visited were graded on a 0 to 10 scale. The CQ‐index was measured at T1.

### 2.6 Statistical methods

#### 2.6.1 Non‐response

Non‐response analysis was performed by comparing respondents with non‐respondents at baseline by patient age, gender and the medical specialty referred to.

#### 2.6.2 Propensity score matching

Since in this cohort study patients were not‐randomly allocated to treatment, patients being referred to PC+ were expected to differ on covariates to those referred to HBOC.^33^, ^34^ To correct for this potential selection bias, which may affect the estimates of the treatment effect, PSM was used.^35^ First, the propensity score (PS) was estimated using logistic regression, which predicts the likelihood of a referral to PC+ or HBOC based on the baseline characteristics described earlier. By matching patients in the intervention and control group based on the PS, the groups will be more balanced on the observed baseline characteristics, which enables to obtain less biased estimates of treatment effects. In this study, one‐to‐two nearest neighbour calliper matching without replacement was used, with a calliper of 0.1.^36^ One‐to‐two matching was used to keep a larger sample size since the HBOC group was small.^37^ Baseline characteristics before and after matching were compared with *P*‐values and standardized mean differences (SMDs). SMDs of <0.1 and *P*‐values of >.05 indicate minor differences in the mean of a covariate between the two groups and were used to assess the success of matching.^38^

#### 2.6.3 Comparing study groups

The overlap in the distribution of the PS and the balance of baseline variables before and after matching between the PC+ and HBOC groups were described.

Marginal linear models with an unstructured error covariance structure were applied to analyse the mean change in HRQoL outcome measures. Estimates, standard errors (SEs), 95% confidence intervals (CIs) and *P*‐values were reported. *P*‐values <.05 were considered as significant. This method takes into account incomplete follow‐up data without any imputation of missing values, and provides valid estimates of treatment effects under the assumption that such data are missing at random.^39^

Patients' experiences of care related items were dichotomized before analysing and summarized as “satisfied” vs “unsatisfied” or “yes” vs “no.” Hereafter, they were analysed using Pearson chi‐square tests; counts, percentages and *P*‐values were reported. Additionally, independent *t*‐tests were used to analyse continuous items; 95% CIs and *P*‐values were reported. To correct for multiple testing (Type 1 error) the Bonferroni correction was used, whereby the *P*‐value of .05 was divided by the number of tests.^40^ Furthermore, analyses were applied without imputation of missing data and items with a high non‐response (more than 10% missing values) were excluded.

Before taking into account the influence of the PS on the HRQoL and experienced quality of care outcome measures using PSM, the uncorrected effect of “study group” was analysed, with “study group” (PC+ vs HBOC) as the only independent variable.^41^

R Studio was used for statistical analyses (R Studio, Boston, MA).

#### 2.6.4 Subgroup analyses

Baseline characteristics, HRQoL and experience of care outcomes before and after PSM were compared between PC+ and HBOC patients separately for the nine different medical specialties using the same analyses as described above.

#### 2.6.5 Sensitivity analyses

Sensitivity analyses were undertaken to assess the robustness of the results.^42^ Analyses were repeated using a one‐to‐one nearest neighbour calliper matching without replacement with a calliper of 0.1.

## 3 RESULTS

### 3.1 Study participants and responders' characteristics

**FIGURE 2:**
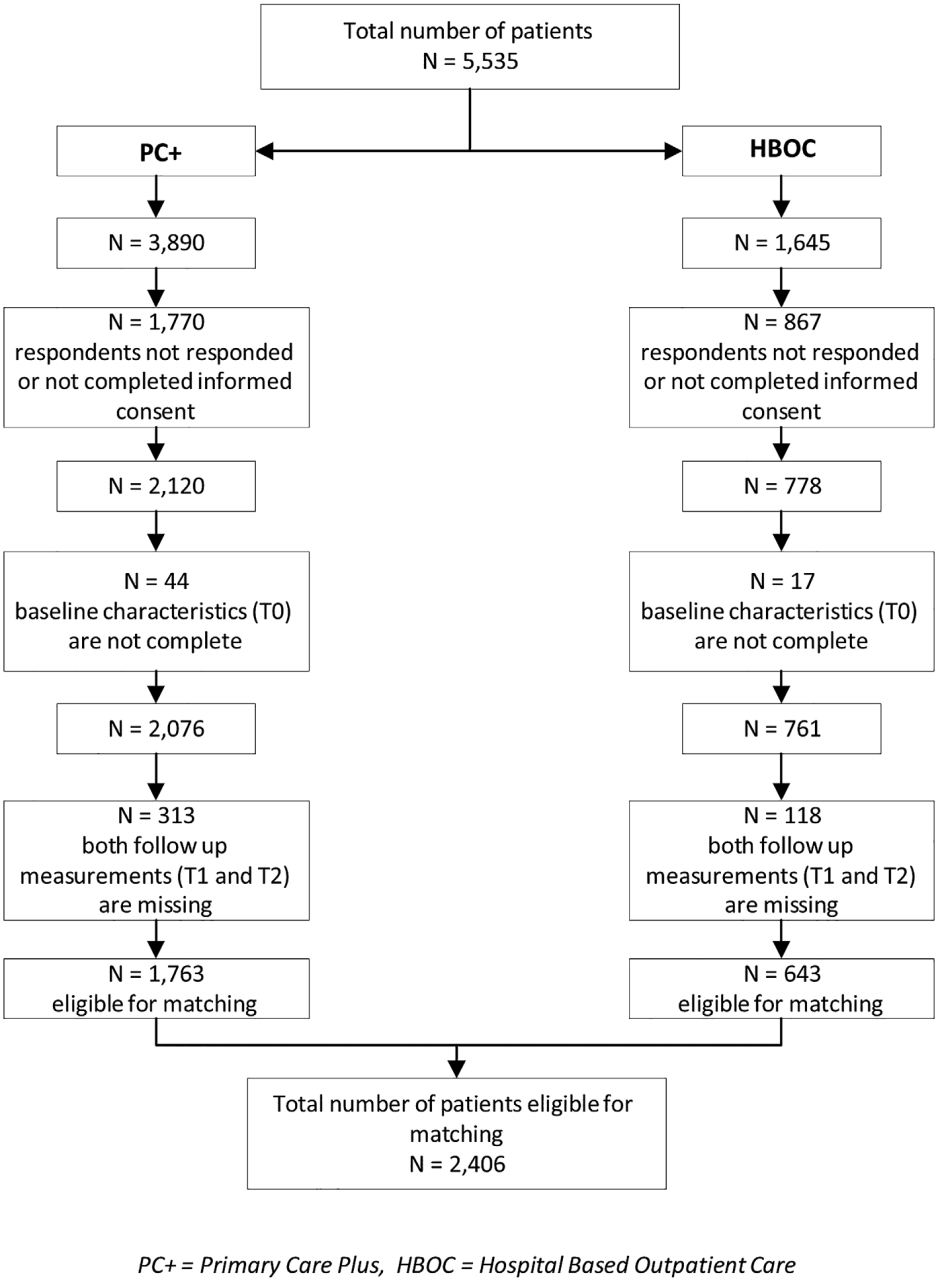
Flow chart of study inclusion

Figure 2 presents a flow chart detailing the inclusion and exclusion of patients. Contact details of 5535 patients were sent to the research team (n = 3890 (70.3%) PC+ group and n = 1645 (29.7%) HBOC group). In total, 2898 patients responded to the informed consent and/or first questionnaire (n = 2120 (54.5%) PC+ group and n = 778 (47.3%) HBOC group). However, the first questionnaire (T0) was not completed by all patients. The first questionnaire was completed by 2076 PC+ patients (53.4%) and 761 HBOC patients (46.3%). Because of missing both follow‐up questionnaires (T1 and T2), 313 (15.1%) PC+ patients and 118 (15.5%) HBOC patients were excluded. As a result, 1763 PC+ patients and 643 HBOC patients were eligible for matching (total N = 2406).

The characteristics of the 2898 responders and 2637 non‐responders are attached in the Table S1. Responders in the PC+ and HBOC group were significantly older compared to non‐responders. Regarding the medical specialty referred to; there was a significant difference in the distribution between responders and non‐responders in the HBOC group, with proportionally more responders referred to ophthalmology, otolaryngology and dermatology.

### 3.2 Inspection for PS overlap before and after matching

**FIGURE 3:**
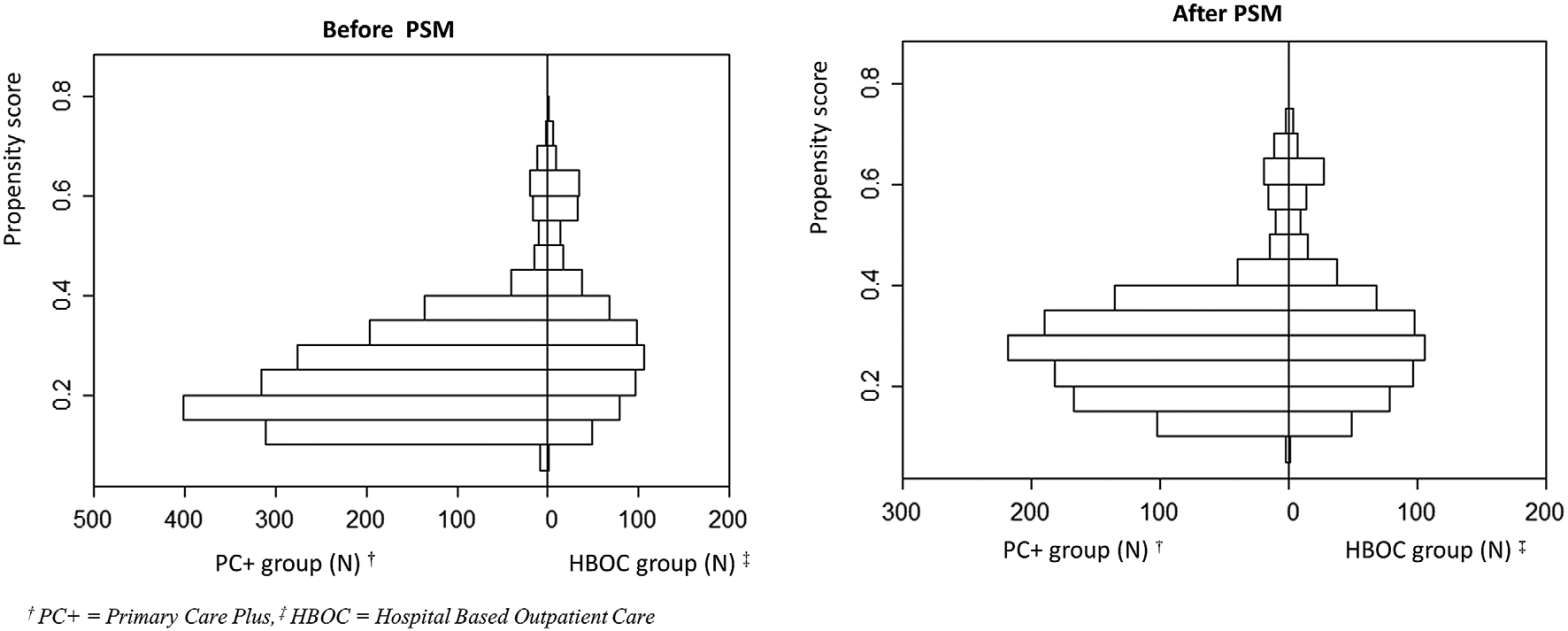
Overlap of the propensity score in the two study groups

Before PSM, the PS for the PC+ group ranged between 0.08 and 0.73; for the HBOC group, the PS ranged between 0.09 and 0.78 (see Figure 3). After PSM, the PS for the PC+ group ranged between 0.10 and 0.73; for the HBOC group, the PS ranged between 0.10 and 0.74.

### 3.3 Baseline characteristics

Prior to PSM, PC+ patients were younger and had a better HRQoL at baseline (Table 1). Furthermore, respectively more PC+ patients were referred to dermatology and rheumatology, and less to internal medicine, neurology, orthopaedics and urology. After PSM, with 1113 PC+ patients matched to 606 HBOC patients, these characteristics were well balanced with a SMD < 0.1, except for the percentage of patients referred to internal medicine (SMD = 0.145).

**TABLE 1:** Baseline characteristics before and after propensity score matching

	Before PSM						After PSM					
	PC+		HBOC		*P*‐value	SMD	PC+		HBOC		*P*‐value	SMD
N	1763		643				1113		606			
Age (mean, SD)	55.95	15.68	57.63	15.23	.019*	0.109	57.88	14.60	57.85	15.13	.960	0.003
Gender (male) (%, SD)	39%	0.49	41%	0.49	.557	0.027	39%	0.49	41%	0.49	.498	0.034
Native country (Netherlands) (%, SD)	97%	0.18	96%	0.20	.393	0.038	96%	0.20	96%	0.20	.876	0.008
Educational level												
Low (%, SD)	19%	0.39	22%	0.42	.063	0.084	21%	0.41	22%	0.42	.511	0.033
Medium (%, SD)	47%	0.50	46%	0.50	.593	0.025	46%	0.50	46%	0.50	.960	0.003
High (%, SD)	34%	0.48	32%	0.47	.317	0.046	33%	0.47	32%	0.47	.531	0.032
EQ‐5D‐5L (mean, SD)	0.81	0.17	0.79	0.17	.005**	0.129	0.79	0.18	0.79	0.17	.896	0.007
EQ‐VAS (mean, SD)	75.53	16.32	73.08	16.31	.001**	0.150	73.34	16.75	73.10	16.37	.767	0.015
SF12 PCS (mean, SD)	47.44	9.33	45.39	10.04	≤.001***	0.211	45.59	9.55	45.37	10.05	.645	0.023
SF12 MCS (mean, SD)	51.22	9.35	50.11	9.34	.010**	0.119	50.77	9.46	50.28	9.34	.302	0.052
BMI (mean, SD)	26.16	4.45	26.44	4.84	.178	0.061	26.53	4.57	26.54	4.82	.961	0.002
Smoking behaviour												
Smoker (%, SD)	16%	0.36	17%	0.38	.443	0.035	16%	0.37	17%	0.38	.591	0.027
Former smoker (%, SD)	42%	0.49	42%	0.49	.771	0.013	42%	0.49	42%	0.49	.948	0.003
Non‐smoker (%, SD)	42%	0.49	41%	0.49	.781	0.013	42%	0.49	41%	0.49	.736	0.017
Alcohol user (%, SD)	62%	0.48	60%	0.49	.198	0.059	60%	0.49	59%	0.49	.810	0.012
Medical specialty referred to												
Dermatology (%, SD)	32%	0.47	16%	0.37	≤.001***	0.371	19%	0.39	17%	0.38	.430	0.040
Gynaecology (%, SD)	5%	0.22	7%	0.25	.260	0.051	7%	0.25	7%	0.25	.992	0.001
Internal medicine (%, SD)	2%	0.15	9%	0.29	<.001***	0.307	4%	0.19	7%	0.25	.003**	0.145
Otolaryngology (%, SD)	17%	0.37	13%	0.34	.046	0.094	16%	0.36	14%	0.35	.454	0.038
Neurology (%, SD)	7%	0.26	12%	0.33	≤.001***	0.159	11%	0.32	12%	0.33	.516	0.033
Ophthalmology (%, SD)	9%	0.28	9%	0.28	.821	0.010	11%	0.31	9%	0.29	.264	0.057
Orthopaedics (%, SD)	19%	0.39	24%	0.43	.009**	0.119	26%	0.44	26%	0.44	.706	0.019
Rheumatology (%, SD)	7%	0.25	4%	0.19	.005**	0.138	4%	0.20	4%	0.20	.728	0.018
Urology (%, SD)	1%	0.12	6%	0.23	≤.001***	0.239	2%	0.15	4%	0.19	.070	0.088

Abbreviations: BMI, body mass index; HBOC, Hospital Based Outpatient Care; PC+, Primary Care Plus; PSM, Propensity score matching; SD, standard deviation; SMD, standardized mean differences.

*Note:* *P < .05; **P < .01; ***P < .001.

### 3.4 Outcome analysis

#### 3.4.1 Health‐related quality of care

Before PSM, the EQ‐5D‐5L baseline score was significantly lower in the HBOC group (*P* < .01) (Table 2). After PSM, the difference at baseline between PC+ and HBOC patients was no longer significant (*P* > .05). Furthermore, the EQ‐5D‐5L scores significantly increased over time (T1 and T2) compared to the baseline score before and after PSM (*P* < .01 or *P* < .001). Finally, after PSM, the interaction terms between time and group were no longer significant, indicating no difference in the development of EQ‐5D‐5L scores between the groups over time (*P* > .05).

**TABLE 2:** Health‐related quality of life outcomes before and after propensity score matching

	Before PSM			After PSM		
EQ‐5D‐5L	Estimate	SE	95%CI	Estimate	SE	95%CI
Intercept	0.82***	0.00	0.81, 0.82	0.79***	0.01	0.78, 0.80
Study group ^a^	−0.02**	0.01	−0.04, −0.01	−0.00.	0.01	−0.02, 0.02
Time T1	0.01***	0.00	0.01, 0.02	0.01**	0.00	0.00, 0.02
Time T2	0.02***	0.00	0.01, 0.02	0.02***	0.00	0.01, 0.02
Time T1 × study group	−0.01*	0.01	−0.02, 0.00	−0.01	0.01	−0.02, 0.00
Time T2 × study group	−0.01*	0.01	−0.03, −0.00	−0.01	0.01	−0.03, 0.00

EQ‐VAS	Estimate	SE	95%CI	Estimate	SE	95%CI
Intercept	75.53***	0.39	74.77, 76.29	73.34***	0.50	72.37, 74.32
Study group ^a^	−2.45**	0.75	−3.92, −0.97	−0.25	0.84	−1.89, 1.40
Time T1	0.92**	0.31	0.32, 1.53	1.11**	0.42	0.29, 1.92
Time T2	1.10	0.64	−0.16, 2.35	1.59	0.91	−0.19, 3.38
Time T1 × study group	−0.41	0.60	−1.58, 0.77	−0.66	0.70	−2.03, 0.72
Time T2 × study group	−0.16	1.25	−2.60, 2.28	−0.62	1.54	−3.64, 2.40

SF‐12 PCS	Estimate	SE	95%CI	Estimate	SE	95%CI
Intercept	47.44***	0.23	46.99, 47.88	45.59***	0.29	45.02, 46.17
Study group ^a^	−2.05***	0.44	−2.91, −1.19	−0.23	0.49	−1.19, 0.74
Time T2	0.57***	0.17	0.24, 0.90	1.02***	0.22	0.59, 1.45
Time T2 × study group	0.11	0.33	−0.53, 0.75	−0.36	0.37	−1.08, 0.37

SF‐12 MCS	Estimate	SE	95%CI	Estimate	SE	95%CI
Intercept	51.22***	0.22	50.78, 51.66	50.77***	0.28	50.21, 51.32
Study group ^a^	−1.11**	0.43	−1.95, −0.27	−0.49	0.48	−1.42, 0.44
Time T2	0.03	0.20	−0.36, 0.42	0.21	0.25	−0.28, 0.71
Time T2 × study group	−0.32	0.39	−1.08, 0.44	−0.65	0.43	−1.49, 0.20

PGIC^b^	Estimate	SE	95%CI	Estimate	SE	95%CI
Intercept	4.57***	0.73	3.14, 6.01	3.45***	0.03	3.40. 3.51
Study group ^a^	−1.10	1.41	−3.88, 1.67	0.03	0.05	−0.06, 0.13
Time T2	−1.58*	0.73	−3.02, −0.15	−0.35***	0.04	−0.43, −0.27
Time T2 × study group	1.11	1.42	−1.67, 3.89	−0.14*	0.07	−0.28, 0.01

Abbreviations: CI, Confidence Interval; HBOC, Hospital Based Outpatient Care; MCS, mental component summary; PC+, Primary Care Plus; PCS, physical component summary; PGIC, Patient Global Impression of Change; PSM, Propensity score matching; SE, Standard Error; T2, 3 months after the consultation.

*Note:* *P < .05; **P < .01; ***P < .001.

^a^ Group was coded as 1 = HBOC group and 0 = PC+ group.

^b^ PGIC was measured at T1 and T2, not at baseline.

Regarding EQ‐VAS outcomes, before PSM, the baseline score was significantly lower in the HBOC group compared to the PC+ group (*P* < .01). After PSM, the difference at baseline was no longer significant (*P* > .05). Furthermore, EQ‐VAS scores significantly increased at T1 compared to the baseline score before and after PSM (*P* < .01). However, no significant interaction terms between time and group were found before and after PSM, indicating no difference in the development of EQ‐VAS scores between the groups over time (*P* > .05).

Regarding SF12v2 scores, before PSM, the PCS and MCS baseline scores were significantly lower in the HBOC group (*P* < .001 and *P* < .01, respectively). After PSM, the differences at baseline were no longer significant (*P* > .05). Furthermore, before and after PSM, the PCS score at T2 was significantly higher compared to the baseline score. However, for both PCS and MCS, no significant interaction terms were found before and after PSM, indicating no difference in the development of the PCS and MCS scores between the groups over time (*P* > .05).

Finally, the PGIC score at T1 did not differ between the PC+ and HBOC groups (*P* > .05). At T2, the PGIC score was significantly lower compared to the score at T1, both before and after PSM (*P* < .05 and *P* < .001, respectively). However, no significant interaction terms between time and group were found before and after PSM, indicating no difference in the development of the PGIC score between the groups over time (*P* > .05).

Figures for the HRQoL outcomes before and after PSM are attached in the Figure S1.

#### 3.4.2 Quality of care

In total, 2365 patients completed the second questionnaire (T1) including the 21 items of the CQ‐index. (n = 1741 PC+ group and n = 624 HBOC group). After PSM, 1681 patients were included in the analysis (n = 1094 PC+ group and n = 587 HBOC group).

One item in the domain of “communication and collaboration between the GP and medical specialist” was excluded from analysis because of high non‐response before (13.5%) and after (13.1%) PSM. Although, only 1230 patients before and 900 patients after PSM completed the item “shared decision‐making,” this item was not excluded since a high number of patients answered “not applicable.” This was the only item in the questionnaire with this answering option. Including the option “not applicable,” 2320 patients (98.1%) completed this item before PSM and 1659 patients (98.7%) after PSM.

Before PSM, PC+ patients significantly more often had a waiting time in the waiting room of less than 30 minutes (*P* ≤ .001) and they gave significantly higher grades to the medical specialist and the PC+ location they visited (*P* = .007 and *P* ≤ .001, respectively) (Table 3). However, after PSM, these differences were no longer significant (*P* = .011, *P* = .199 and *P* = .354, respectively). Furthermore, before PSM, the travel time to the PC+ or HBOC location was significantly shorter in the PC+ group (*P* ≤ .001). However, after PSM, the travel time was significantly shorter in the HBOC group (*P* ≤ .001).

**TABLE 3:** Comparison of patient‐experienced quality of care outcomes before and after propensity score matching

	Before PSM			After PSM		
	PC+	HBOC	*P*‐value	PC+	HBOC	*P*‐value
N	1741	624		1094	587	
Quality of care domains	Satisfied/Yes n (%)	Satisfied/Yes n (%)		Satisfied/Yes n (%)	Satisfied/Yes n (%)	
Timeliness (1)						
Waiting time for appointment	89.2(1527)	86.0(533)	.032	89.9 (972)	86.5(505)	.034
Waiting time in waiting room <30 minutes	93.5 (1605)	88.5 (546)	≤.001*	92.0 (997)	88.1(513)	.011
Treatment by the medical specialist						
Complaint was taken seriously	97.5(1672)	97.4(601)	.845	97.0(1052)	97.3(566)	.813
Specialist listened carefully	97.3(1667)	97.4(601)	.845	96.9(1050)	97.3(566)	.659
Specialist took enough time	98.0(1679)	98.7(608)	.240	97.9(1061)	98.6(573)	.284
Treated with respect	98.8(1692)	98.5(607)	.574	98.4(1066)	98.5(572)	.974
Competence of the specialist	98.4(1673)	98.0(601)	.543	98.5(1063)	98.1(567)	.520
Overall help of the specialist	94.2(1612)	93.5(575)	.553	94.0(1018)	93.3(541)	.562
Information provision and communication by the medical specialist						
Information about different treatment options	92.6(1581)	90.7(555)	.140	92.2(998)	90.5(523)	.220
Understandable explanation	97.1(1663)	96.1(592)	.236	97.4(1055)	95.9(557)	.084
Opportunity to ask questions	97.4(1666)	96.4(594)	.231	97.2(1055)	95.9(557)	.256
Shared decision‐making	88.4(892)	87.3(338)	.582	88.3(580)	87.0(320)	.535
Communication and collaboration between the GP and medical specialist						
Matching recommendations between GP and specialist	80.3(1357)	82.1(501)	.324	79.1(846)	82.1(472)	.153
Awareness of the medical specialist about the complaint	89.4(1519)	89.1(547)	.827	88.1(946)	89.5(518)	.399
Collaboration and alignment between GP and specialist	85.8(1366)	81.6(482)	.016	85.2(859)	81.7(454)	.066
Overall assessment of quality of care (1)						
Recommend medical specialist to family/friends	93.7(1598)	92.5(568)	.298	92.9(1002)	92.2(544)	.506
Recommend PC+/HBOC to family/friends	95.4(1625)	93.8(577)	.119	95.1(1024)	93.8(544)	.268

	Mean (SD)	Mean (SD)		Mean (SD)	Mean (SD)	
Timeliness (2)						
Travel time (in minutes) ^a^	15.6 (9.34)	19.2 (12.18)	≤.001*	17.0 (10.75)	15.6 (9.10)	.001*
Overall assessment of quality of care (2)						
Grade specialist (0–10)	8.5 (1.15)	8.4 (1.22)	.007	8.5 (1.15)	8.5 (1.20)	.199
Grade PC+/HBOC (0‐10)	8.5 (1.08)	8.3 (1.11)	≤.001*	8.4 (1.08)	8.5 (1.12)	.354

Abbreviations: HBOC, Hospital Based Outpatient Care; PC+, Primary Care Plus; PSM, Propensity score matching; SD, Standard Deviation.

* *P* < .0025 were considered as significant according to the Bonferroni correction.

^a^ A significant higher score on travel time means a longer travel time in minutes and is in this case an unfavourable result.

#### 3.4.3 Subgroup analyses

In the subgroup analyses, the baseline characteristics, HRQoL and experiences of care related outcomes before and after PSM were analysed per medical specialty. Regarding baseline characteristics, all medical specialties had two or more characteristics with a SMD > 0.1, indicating less balanced groups (see Table S3).

Regarding HRQoL outcomes, significant interactions between time and group after PSM were found for the medical specialties neurology, otolaryngology and internal medicine, indicating a positive effect for PC+ patients over time (see Table S3). Time effects were found for neurology on the EQ‐5D‐5L at T1 and on the EQ‐VAS at T1 and T2, for otolaryngology on the SF12v2 MCS and the PGIC, and for internal medicine on the SF12v2 MCS. However, for dermatology, a negative effect was found on the SF12v2 PCS score, indicating that HBOC resulted in better outcomes on the physical component over time compared to PC+.

Regarding experienced quality of care outcomes measured on 20 items, after PSM PC+ scored higher on three items for dermatology and on one item for neurology (see Table S4). Furthermore, a significantly higher score on travel time (meaning a longer travel time) was found for HBOC patients referred to dermatology, otolaryngology and orthopaedics.

#### 3.4.4 Sensitivity analyses

After one‐to‐one PSM, the PS for the PC+ group ranged between 0.09 and 0.73; for the HBOC group, the PS ranged between 0.10 and 0.74 (see Figure S2). In total, 609 PC+ patients were matched to 609 HBOC patients with well‐balanced baseline characteristics (all SMD < 0.1 and *P*‐values > .05; see Table S5). Regarding HRQoL outcome analysis after one‐to‐one PSM, the results were comparable to one‐to‐two PSM with no significant interaction terms between time and group (see Table S6). Regarding experienced quality of care after one‐to‐one PSM, most results were comparable to one‐to‐two PSM (see Table S7). However, the difference in travel time to the PC+ or HBOC location was no longer significant (*P* = .212).

## 4 DISCUSSION

In this study, PSM resulted in balanced groups with respect to measured baseline characteristics. Therefore, a better comparison could be made between the effects of PC+ and HBOC on the health of the population and patients' experiences of care. The results showed that PC+ care for low‐complex and non‐acute patients delivered in a primary care setting without the presence of hospital facilities led to the maintenance of patients' experiences of HRQoL and quality of care.

These results are generally consistent to those of Quanjel et al^17^ who evaluated a PC+ intervention focusing on cardiologists providing consultations in a primary care setting. They concluded that PC+ results in equal effects on HRQoL outcomes over time and improved quality of care as experienced by patients compared to care as‐usual. Other studies including shifted HBOC also found high levels of patient satisfaction.^43^, ^44^

This study showed positive results regarding patients' experiences of HRQoL in PC+. To measure HRQoL, generic instruments were used since they are applicable to all patients, regardless of the medical specialty referred to and regardless of the patient's condition. Therefore, comparison between different medical specialties and interventions is possible.^45^ However, generic instruments are limited in detecting change over time (responsiveness) compared to disease‐ or condition‐specific instruments.^46^ Therefore, equal effect on HRQoL outcomes could be the result of the use of generic instruments to measure the HRQoL over time. In future research, using both generic and condition‐specific instruments should be considered to increase responsiveness.

Furthermore, this study showed that patients were highly satisfied with the care delivered in PC+. This is a positive result, although it is recognized that patients remain reluctant to be critical about the care they receive.^47^ This is based on patient desire to be grateful, as well as their recognition of the inevitable limitations of health care. However, patient satisfaction could be supplemented with clinical outcome measures focused on effectiveness and appropriateness of care, to provide vital feedback for improvements if necessary. In addition, the shorter travel time to HBOC can be explained because HBOC is more accessible, for example by public transportation, compared to the PC+ locations. Although PC+ focuses on care delivered closer to patients' homes, this does not guarantee a shorter travel time. This can be important for patients who rely on public transportation.^48^

Despite the estimated PS balanced covariates for the overall study population, subgroups based on medical specialty showed large variability in covariates. Therefore, caution is advised in the interpretation of the HRQoL and experienced quality of care outcomes per medical specialty. Instead of a cohort study, a randomized controlled trial (RCT) with block randomization could be a useful technique to achieve balance in the allocation of patients to subgroups and therewith reduce bias.^49^ However, performing an RCT in this case was not possible and not preferable since the PC+ intervention was subject to change during the study period, with inflow and outflow of medical specialties, for example. Furthermore, an important principle in this intervention was that GPs remain responsible for the patient and therefore they decided in agreement with the patient whether to refer a patient to PC+.

There are several limitations to this study. Although PSM permits a more objective analysis by balancing the study groups with respect to confounders, it only allows for adjustment of measured confounders.^37^ However, this limitation is applicable for all datasets and all multivariable adjustment methods. Sensitivity analysis was performed to assess the robustness of the study results. As the results changed minimally regarding statistical significance and direction of the association, confidence was provided that no significant unmeasured baseline characteristics were influencing the PC+ effect.^50^ Only travel time turned out to be sensitive to the PSM method used. Furthermore, this study seems to be affected by non‐responder bias since non‐responders turned out to be significantly younger compared to responders.^51^ Finally, this study was based on a single region with one primary care organization and one hospital, which limits the generalizability of the results.

In conclusion, this study found equal results on HRQoL and experienced quality of care outcomes between patients referred to PC+ and HBOC. Therefore, it can be concluded that, despite the lack of diagnostic tools, population health and quality of care are maintained in PC+. In future research, there should be more emphasis on cost comparison for patients and for the total health system to demonstrate the potential added value of PC+.

## CONFLICT OF INTEREST

The authors declare no conflict of interest.

## Supporting information


**TABLE S1.** Baseline Characteristics of Responders and Non‐Responders.

**TABLE S2.** Baseline Characteristics Before and After Propensity Score Matching per Medical Specialty.

**TABLE S3.** Health‐Related Quality of Life Outcomes Before and After Propensity Score Matching per Medical Specialty.

**TABLE S4.** Comparison of Patient Experienced Quality of Care Outcomes Before and After Propensity Score Matching per Medical Specialty.

**TABLE S5.** Baseline Characteristics Before and After Propensity Score Matching.

**TABLE S6.** Health‐Related Quality of Life Outcomes Before and After Propensity Score Matching.

**TABLE S7.** Comparison of Patient Experienced Quality of Care Outcomes Before and After Propensity Score Matching.

**FIGURE S1.** Uncorrected and corrected health‐related quality of life outcomes.

**FIGURE S2.** Overlap of the Propensity Score in the Two Study Groups.
