# Supplementary material for: Patients' perspectives on a new delivery model in primary care: A propensity score matched analysis of patient‐reported outcomes in a Dutch cohort study
Source: J Eval Clin Pract. 2020 Jun 17;27(2):344–55. doi: 10.1111/jep.13426 (PMC7983912; doi:10.1111/jep.13426)
Supplement: Supplementary file 1 — TABLE S1. Baseline Characteristics of Responders and Non‐Responders. [file JEP-27-344-s005.docx]

**Table S1** Baseline Characteristics of Responders and Non-Responders

|  | **PC+** | |  | | **HBOC** | |  |
| --- | --- | --- | --- | --- | --- | --- | --- |
| **Characteristics** | **Responders** | **Non-responders** | | **P-value** | **Responders** | **Non-responders** | **P-value** |
| N (%) | 2,120 (54.5) | 1,770 (45.5) | |  | 778 (47.3) | 867 (52.7) |  |
| Age (mean, SD) ^†^ | 54.6 (16.32) | 48.1 (17.06) | | ≤ 0.001^*^ | 56.5 (15.79) | 49.73 (17.34) | ≤ 0.001^*^ |
| Gender (male) N (%) ^‡^ | 721 (40.8) | 817 (38.5) | | 0.159 | 337 (38.9) | 314 (40.4) | 0.537 |
| Medical specialty referred to ^§^ |  |  | | 0.716 |  |  | ≤ 0.001^*^ |
| Dermatology N (%) | 661 (31.2) | 565 (32.1) | |  | 127 (16.3) | 119 (13.8) |  |
| Gynaecology N (%) | 126 (5.9) | 92 (5.2) | |  | 55 (7.1) | 102 (11.8) |  |
| Internal medicine N (%) | 58 (2.7) | 55 (3.1) | |  | 71 (9.1) | 91 (10.6) |  |
| Otolaryngology N (%) | 359 (16.9) | 316 (18.0) | |  | 103 (13.2) | 88 (10.2) |  |
| Neurology N (%) | 158 (7.5) | 121 (6.9) | |  | 96 (12.3) | 148 (17.2) |  |
| Ophthalmology N (%) | 178 (8.4) | 144 (8.2) | |  | 62 (8.0) | 42 (4.9) |  |
| Orthopaedics N (%) | 402 (19.0) | 316 (18.0) | |  | 188 (24.2) | 205 (23.8) |  |
| Rheumatology N (%) | 142 (6.7) | 132 (7.5) | |  | 30 (3.9) | 28 (3.3) |  |
| Urology N (%) | 26 (1.2) | 15 (0.9) | |  | 46 (5.9) | 38 (4.4) |  |

*PC+ = Primary Care Plus; HBOC = Hospital Based Outpatient Care; SD= Standard deviation*

*^†^Age at date of appointment was missing for n=69 patients (PC+ group: n=2 non-responders and HBOC group: n=1 responders and n=66 non-responders);*  *^‡^Gender of n=1 patient was missing (PC+ group: n=1 responder); ^§^ Medical specialty referred to was missing for n=16 patients (PC+ group: n=10 non-responders and HBOC group: n= 6 non-responders)*

** P < 0.001*
