# Supplementary material for: Patients' perspectives on a new delivery model in primary care: A propensity score matched analysis of patient‐reported outcomes in a Dutch cohort study
Source: J Eval Clin Pract. 2020 Jun 17;27(2):344–55. doi: 10.1111/jep.13426 (PMC7983912; doi:10.1111/jep.13426)
Supplement: Supplementary file 2 — TABLE S2. Baseline Characteristics Before and After Propensity Score Matching per Medical Specialty. [file JEP-27-344-s009.docx]

**Table S2** Baseline Characteristics Before and After Propensity Score Matching per Medical Specialty

| **Dermatology** | | **Before PSM** | | | | | | | | | | | | **After PSM** | | | | | | | | | | | |
| --- | --- | --- | --- | --- | --- | --- | --- | --- | --- | --- | --- | --- | --- | --- | --- | --- | --- | --- | --- | --- | --- | --- | --- | --- | --- |
|  |  | **PC+** | | | | **HBOC** | | | | **P-value** | | **SMD** | | **PC+** | | | | **HBOC** | | | | **P-value** | | **SMD** | |
| N | | 563 | | | | 105 | | | |  | |  | | 210 | | | | 105 | | | |  | |  | |
| Age (mean, SD) | | 54.85 | | 16.83 | | 56.96 | | 16.72 | | 0.238 | | 0.126 | | 58.4 | | 15.55 | | 56.96 | | 16.72 | | 0.453 | | 0.089 | |
| Gender (male) (%, SD) | | 39% | | 0.49 | | 44% | | 0.50 | | 0.382 | | 0.092 | | 38% | | 0.49 | | 44% | | 0.50 | | 0.291 | | 0.126 | |
| Native country (Netherlands) (%, SD) | | 97% | | 0.16 | | 91% | | 0.28 | | 0.003** | | 0.258 | | 97% | | 0.17 | | 91% | | 0.28 | | 0.025* | | 0.247 | |
| Educational level | |  | |  | |  | |  | |  | |  | |  | |  | |  | |  | |  | |  | |
| Low (%, SD) | | 18% | | 0.39 | | 20% | | 0.40 | | 0.648 | | 0.048 | | 21% | | 0.41 | | 20% | | 0.40 | | 0.770 | | 0.035 | |
| Medium (%, SD) | | 44% | | 0.50 | | 49% | | 0.50 | | 0.432 | | 0.083 | | 46% | | 0.50 | | 49% | | 0.50 | | 0.691 | | 0.048 | |
| High (%, SD) | | 37% | | 0.48 | | 31% | | 0.47 | | 0.238 | | 0.127 | | 32% | | 0.47 | | 31% | | 0.47 | | 0.865 | | 0.020 | |
| EQ-5D-5L (mean, SD) | | 0.88 | | 0.14 | | 0.86 | | 0.17 | | 0.29 | | 0.104 | | 79.19 | | 14.22 | | 77.05 | | 17.18 | | 0.242 | | 0.136 | |
| EQ VAS (mean, SD) | | 81.02 | | 13.36 | | 77.05 | | 17.18 | | 0.008** | | 0.258 | | 0.86 | | 0.14 | | 0.86 | | 0.17 | | 0.901 | | 0.014 | |
| SF12 PCS (mean, SD) | | 51.24 | | 7.80 | | 48.87 | | 10.43 | | 0.007** | | 0.257 | | 51.93 | | 8.94 | | 51.4 | | 9.20 | | 0.623 | | 0.058 | |
| SF12 MCS (mean, SD) | | 52.05 | | 8.75 | | 51.4 | | 9.20 | | 0.485 | | 0.073 | | 49.62 | | 8.43 | | 48.87 | | 10.43 | | 0.497 | | 0.078 | |
| BMI (mean, SD) | | 25.46 | | 4.19 | | 26.56 | | 5.37 | | 0.019* | | 0.228 | | 25.6 | | 4.49 | | 26.56 | | 5.37 | | 0.096 | | 0.193 | |
| Smoking behaviour | |  | |  | |  | |  | |  | |  | |  | |  | |  | |  | |  | |  | |
| Smoker (%, SD) | | 15% | | 0.36 | | 16% | | 0.37 | | 0.849 | | 0.020 | | 20% | | 0.40 | | 16% | | 0.37 | | 0.473 | | 0.087 | |
| Former smoker (%, SD) | | 42% | | 0.49 | | 44% | | 0.50 | | 0.669 | | 0.045 | | 42% | | 0.49 | | 44% | | 0.50 | | 0.748 | | 0.038 | |
| Non-smoker (%, SD) | | 43% | | 0.50 | | 40% | | 0.49 | | 0.571 | | 0.060 | | 39% | | 0.49 | | 40% | | 0.49 | | 0.807 | | 0.029 | |
| Alcohol user (%, SD) | | 63% | | 0.48 | | 64% | | 0.48 | | 0.938 | | 0.008 | | 59% | | 0.49 | | 64% | | 0.48 | | 0.372 | | 0.107 | |
| **Gynaecology** | | **Before PSM** | | | | | | | | | | | | **After PSM** | | | | | | | | | | | |
|  |  | **PC+** | | | | **HBOC** | | | | **P-value** | | **SMD** | | **PC+** | | | | **HBOC** | | | | **P-value** | | **SMD** | |
| N | | 94 | | | | 42 | | | |  | |  | | 77 | | | | 42 | | | |  | |  | |
| Age (mean, SD) | | 41.54 | | 16.38 | | 41.86 | | 13.81 | | 0.914 | | 0.021 | | 44.51 | | 15.77 | | 41.86 | | 13.81 | | 0.363 | | 0.179 | |
| Gender (male) (%, SD) | | 0% | | 0.00 | | 0% | | 0.00 | | - | | - | | 0% | | 0.00 | | 0% | | 0.00 | | - | | - | |
| Native country (Netherlands) (%, SD) | | 91% | | 0.28 | | 98% | | 0.15 | | 0.187 | | 0.271 | | 92% | | 0.27 | | 98% | | 0.15 | | 0.234 | | 0.246 | |
| Educational level | |  | |  | |  | |  | |  | |  | |  | |  | |  | |  | |  | |  | |
| Low (%, SD) | | 4% | | 0.20 | | 17% | | 0.38 | | 0.014* | | 0.410 | | 5% | | 0.22 | | 17% | | 0.38 | | 0.039* | | 0.37 | |
| Medium (%, SD) | | 56% | | 0.50 | | 31% | | 0.47 | | 0.006** | | 0.526 | | 55% | | 0.50 | | 31% | | 0.47 | | 0.013* | | 0.487 | |
| High (%, SD) | | 39% | | 0.49 | | 52% | | 0.51 | | 0.159 | | 0.261 | | 40% | | 0.49 | | 52% | | 0.51 | | 0.207 | | 0.243 | |
| EQ-5D-5L (mean, SD) | | 0.89 | | 0.13 | | 0.85 | | 0.20 | | 0.137 | | 0.255 | | 0.89 | | 0.14 | | 0.85 | | 0.20 | | 0.189 | | 0.238 | |
| EQ VAS (mean, SD) | | 82.88 | | 13.76 | | 80.17 | | 13.55 | | 0.287 | | 0.199 | | 82.21 | | 13.89 | | 80.17 | | 13.55 | | 0.441 | | 0.149 | |
| SF12 PCS (mean, SD) | | 52.49 | | 8.71 | | 52.24 | | 7.80 | | 0.87 | | 0.031 | | 51.36 | | 9.13 | | 52.24 | | 7.80 | | 0.601 | | 0.103 | |
| SF12 MCS (mean, SD) | | 51.03 | | 9.45 | | 47.39 | | 9.00 | | 0.037* | | 0.394 | | 50.85 | | 9.63 | | 47.39 | | 9.00 | | 0.058 | | 0.371 | |
| BMI (mean, SD) | | 25.42 | | 4.50 | | 24.73 | | 3.67 | | 0.386 | | 0.167 | | 25.91 | | 4.72 | | 24.73 | | 3.67 | | 0.161 | | 0.281 | |
| Smoking behaviour | |  | |  | |  | |  | |  | |  | |  | |  | |  | |  | |  | |  | |
| Smoker (%, SD) | | 12% | | 0.32 | | 21% | | 0.42 | | 0.141 | | 0.261 | | 13% | | 0.34 | | 21% | | 0.42 | | 0.233 | | 0.223 | |
| Former smoker (%, SD) | | 22% | | 0.42 | | 26% | | 0.45 | | 0.628 | | 0.089 | | 26% | | 0.44 | | 26% | | 0.45 | | 0.980 | | 0.005 | |
| Non-smoker (%, SD) | | 66% | | 0.48 | | 52% | | 0.51 | | 0.134 | | 0.276 | | 61% | | 0.49 | | 52% | | 0.51 | | 0.365 | | 0.174 | |
| Alcohol user (%, SD) | | 61% | | 0.49 | | 60% | | 0.50 | | 0.903 | | 0.023 | | 56% | | 0.50 | | 60% | | 0.50 | | 0.701 | | 0.074 | |
| **Otolaryngology** | | **Before PSM** | | | | | | | | | | | | **After PSM** | | | | | | | | | | | |
|  |  | **PC+** | | | | **HBOC** | | | | **P-value** | | **SMD** | | **PC+** | | | | **HBOC** | | | | **P-value** | | **SMD** | |
| N | | 295 | | | | 86 | | | |  | |  | | 173 | | | | 86 | | | |  | |  | |
| Age (mean, SD) | | 58.73 | | 14.25 | | 59.07 | | 14.14 | | 0.845 | | 0.024 | | 61.17 | | 12.38 | | 59.07 | | 14.14 | | 0.222 | | 0.086 | |
| Gender (male) (%, SD) | | 51% | | 0.50 | | 43% | | 0.50 | | 0.203 | | 0.157 | | 50% | | 0.50 | | 43% | | 0.50 | | 0.272 | | 0.124 | |
| Native country (Netherlands) (%, SD) | | 97% | | 0.17 | | 98% | | 0.15 | | 0.725 | | 0.045 | | 97% | | 0.18 | | 98% | | 0.15 | | 0.618 | | 0.159 | |
| Educational level | |  | |  | |  | |  | |  | |  | |  | |  | |  | |  | |  | |  | |
| Low (%, SD) | | 21% | | 0.41 | | 19% | | 0.39 | | 0.627 | | 0.060 | | 24% | | 0.43 | | 19% | | 0.39 | | 0.353 | | 0.090 | |
| Medium (%, SD) | | 42% | | 0.49 | | 35% | | 0.48 | | 0.258 | | 0.140 | | 35% | | 0.48 | | 35% | | 0.48 | | 0.953 | | 0.025 | |
| High (%, SD) | | 37% | | 0.48 | | 47% | | 0.50 | | 0.124 | | 0.187 | | 41% | | 0.49 | | 47% | | 0.50 | | 0.404 | | 0.100 | |
| EQ-5D-5L (mean, SD) | | 0.84 | | 0.14 | | 0.84 | | 0.12 | | 0.809 | | 0.031 | | 0.84 | | 0.13 | | 0.84 | | 0.12 | | 0.933 | | 0.052 | |
| EQ VAS (mean, SD) | | 76.22 | | 16.12 | | 72.77 | | 16.32 | | 0.082 | | 0.213 | | 74.97 | | 16.48 | | 72.77 | | 16.32 | | 0.311 | | 0.004 | |
| SF12 PCS (mean, SD) | | 49.07 | | 8.57 | | 48.05 | | 7.39 | | 0.319 | | 0.127 | | 48.05 | | 8.81 | | 48.05 | | 7.39 | | 0.996 | | 0.069 | |
| SF12 MCS (mean, SD) | | 51.02 | | 8.97 | | 49.86 | | 9.57 | | 0.297 | | 0.126 | | 50.17 | | 9.31 | | 49.86 | | 9.57 | | 0.799 | | 0.023 | |
| BMI (mean, SD) | | 26.11 | | 4.29 | | 26.16 | | 4.67 | | 0.929 | | 0.011 | | 26.31 | | 4.26 | | 26.16 | | 4.67 | | 0.789 | | 0.036 | |
| Smoking behaviour | |  | |  | |  | |  | |  | |  | |  | |  | |  | |  | |  | |  | |
| Smoker (%, SD) | | 14% | | 0.35 | | 10% | | 0.31 | | 0.367 | | 0.114 | | 12% | | 0.33 | | 10% | | 0.31 | | 0.693 | | 0.040 | |
| Former smoker (%, SD) | | 45% | | 0.50 | | 47% | | 0.50 | | 0.773 | | 0.035 | | 43% | | 0.50 | | 47% | | 0.50 | | 0.570 | | 0.001 | |
| Non-smoker (%, SD) | | 41% | | 0.49 | | 43% | | 0.50 | | 0.740 | | 0.041 | | 45% | | 0.50 | | 43% | | 0.50 | | 0.754 | | 0.025 | |
| Alcohol user (%, SD) | | 69% | | 0.46 | | 60% | | 0.49 | | 0.148 | | 0.175 | | 66% | | 0.47 | | 60% | | 0.49 | | 0.343 | | 0.076 | |

| **Internal medicine** | **Before PSM** | | | | | | **After PSM** | | | | | |
| --- | --- | --- | --- | --- | --- | --- | --- | --- | --- | --- | --- | --- |
|  | **PC+** | | **HBOC** | | **P-value** | **SMD** | **PC+** | | **HBOC** | | **P-value** | **SMD** |
| N | 41 | | 61 | |  |  | 41 | | 42 | |  |  |
| Age (mean, SD) | 52.54 | 18.46 | 54.74 | 16.19 | 0.526 | 0.127 | 52.54 | 18.46 | 55.81 | 15.09 | 0.379 | 0.194 |
| Gender (male) (%, SD) | 34% | 0.48 | 36% | 0.48 | 0.844 | 0.040 | 34% | 0.48 | 43% | 0.50 | 0.421 | 0.178 |
| Native country (Netherlands) (%, SD) | 98% | 0.16 | 98% | 0.13 | 0.778 | 0.056 | 98% | 0.16 | 100% | 0.00 | 0.314 | 0.221 |
| Educational level |  |  |  |  |  |  |  |  |  |  |  |  |
| Low (%, SD) | 22% | 0.42 | 13% | 0.34 | 0.245 | 0.231 | 22% | 0.42 | 10% | 0.30 | 0.122 | 0.342 |
| Medium (%, SD) | 46% | 0.50 | 57% | 0.50 | 0.278 | 0.220 | 46% | 0.50 | 64% | 0.48 | 0.102 | 0.363 |
| High (%, SD) | 32% | 0.47 | 30% | 0.46 | 0.815 | 0.047 | 32% | 0.47 | 26% | 0.45 | 0.585 | 0.120 |
| EQ-5D-5L (mean, SD) | 0.74 | 0.22 | 0.78 | 0.14 | 0.244 | 0.227 | 0.74 | 0.22 | 0.79 | 0.13 | 0.226 | 0.267 |
| EQ VAS (mean, SD) | 67.63 | 17.37 | 70.03 | 15.90 | 0.473 | 0.144 | 67.63 | 17.37 | 69.93 | 16.35 | 0.537 | 0.136 |
| SF12 PCS (mean, SD) | 43.93 | 10.56 | 45.84 | 9.89 | 0.355 | 0.187 | 43.93 | 10.56 | 45.74 | 10.44 | 0.435 | 0.172 |
| SF12 MCS (mean, SD) | 46.28 | 10.28 | 48.57 | 10.33 | 0.274 | 0.222 | 46.28 | 10.28 | 49.64 | 10.70 | 0.148 | 0.320 |
| BMI (mean, SD) | 26.28 | 3.88 | 25.36 | 4.65 | 0.299 | 0.215 | 26.28 | 3.88 | 25.7 | 4.40 | 0.526 | 0.140 |
| Smoking behaviour |  |  |  |  |  |  |  |  |  |  |  |  |
| Smoker (%, SD) | 12% | 0.33 | 21% | 0.41 | 0.241 | 0.244 | 12% | 0.33 | 19% | 0.40 | 0.397 | 0.187 |
| Former smoker (%, SD) | 37% | 0.49 | 36% | 0.48 | 0.958 | 0.011 | 37% | 0.49 | 38% | 0.49 | 0.889 | 0.031 |
| Non-smoker (%, SD) | 51% | 0.51 | 43% | 0.50 | 0.398 | 0.171 | 51% | 0.51 | 43% | 0.50 | 0.451 | 0.166 |
| Alcohol user (%, SD) | 63% | 0.49 | 52% | 0.50 | 0.278 | 0.221 | 63% | 0.49 | 48% | 0.51 | 0.151 | 0.318 |
| **Neurology** | **Before PSM** | | | | | | **After PSM** | | | | | |
|  | **PC+** | | **HBOC** | | **P-value** | **SMD** | **PC+** | | **HBOC** | | **P-value** | **SMD** |
| N | 131 | | 78 | |  |  | 126 | | 75 | |  |  |
| Age (mean, SD) | 55.04 | 15.12 | 58.91 | 15.35 | 0.076 | 0.254 | 55.77 | 14.84 | 58.36 | 15.40 | 0.239 | 0.171 |
| Gender (male) (%, SD) | 42% | 0.50 | 46% | 0.50 | 0.559 | 0.084 | 43% | 0.50 | 45% | 0.50 | 0.734 | 0.050 |
| Native country (Netherlands) (%, SD) | 96% | 0.19 | 96% | 0.19 | 0.992 | 0.002 | 96% | 0.20 | 96% | 0.20 | 0.991 | 0.002 |
| Educational level |  |  |  |  |  |  |  |  |  |  |  |  |
| Low (%, SD) | 17% | 0.38 | 31% | 0.46 | 0.018 | 0.331 | 17% | 0.38 | 28% | 0.45 | 0.079 | 0.252 |
| Medium (%, SD) | 53% | 0.50 | 41% | 0.50 | 0.104 | 0.234 | 51% | 0.50 | 43% | 0.50 | 0.267 | 0.163 |
| High (%, SD) | 31% | 0.46 | 28% | 0.45 | 0.723 | 0.051 | 32% | 0.47 | 29% | 0.46 | 0.722 | 0.052 |
| EQ-5D-5L (mean, SD) | 0.71 | 0.22 | 0.76 | 0.14 | 0.099 | 0.250 | 0.71 | 0.23 | 0.76 | 0.14 | 0.078 | 0.273 |
| EQ VAS (mean, SD) | 65.7 | 17.66 | 70.15 | 12.97 | 0.054 | 0.287 | 65.59 | 17.83 | 70.47 | 12.88 | 0.040* | 0.314 |
| SF12 PCS (mean, SD) | 43.18 | 8.93 | 42.67 | 9.04 | 0.693 | 0.057 | 42.98 | 9.02 | 43.04 | 8.90 | 0.961 | 0.007 |
| SF12 MCS (mean, SD) | 48.76 | 10.19 | 49.94 | 9.44 | 0.409 | 0.119 | 48.43 | 10.23 | 50.13 | 9.56 | 0.243 | 0.172 |
| BMI (mean, SD) | 26.4 | 4.76 | 26.52 | 4.45 | 0.857 | 0.026 | 26.45 | 4.79 | 26.55 | 4.47 | 0.881 | 0.022 |
| Smoking behaviour |  |  |  |  |  |  |  |  |  |  |  |  |
| Smoker (%, SD) | 19% | 0.39 | 21% | 0.41 | 0.803 | 0.036 | 20% | 0.40 | 21% | 0.41 | 0.801 | 0.037 |
| Former smoker (%, SD) | 46% | 0.50 | 40% | 0.49 | 0.395 | 0.122 | 46% | 0.50 | 40% | 0.49 | 0.407 | 0.121 |
| Non-smoker (%, SD) | 35% | 0.48 | 40% | 0.49 | 0.505 | 0.095 | 34% | 0.48 | 39% | 0.49 | 0.519 | 0.094 |
| Alcohol user (%, SD) | 59% | 0.49 | 62% | 0.49 | 0.696 | 0.056 | 58% | 0.50 | 61% | 0.49 | 0.638 | 0.069 |
| **Ophthalmology** | **Before PSM** | | | | | | **After PSM** | | | | | |
|  | **PC+** | | **HBOC** | | **P-value** | **SMD** | **PC+** | | **HBOC** | | **P-value** | **SMD** |
| N | 156 | | 55 | |  |  | 120 | | 55 | |  |  |
| Age (mean, SD) | 62.57 | 12.55 | 63.20 | 11.36 | 0.744 | 0.053 | 63.72 | 11.87 | 63.20 | 11.36 | 0.787 | 0.044 |
| Gender (male) (%, SD) | 44% | 0.50 | 42% | 0.50 | 0.821 | 0.036 | 45% | 0.50 | 42% | 0.50 | 0.696 | 0.064 |
| Native country (Netherlands) (%, SD) | 96% | 0.19 | 96% | 0.19 | 0.944 | 0.011 | 95% | 0.22 | 96% | 0.19 | 0.691 | 0.067 |
| Educational level |  |  |  |  |  |  |  |  |  |  |  |  |
| Low (%, SD) | 21% | 0.41 | 24% | 0.43 | 0.629 | 0.075 | 22% | 0.42 | 24% | 0.43 | 0.869 | 0.027 |
| Medium (%, SD) | 46% | 0.50 | 40% | 0.49 | 0.481 | 0.111 | 45% | 0.50 | 40% | 0.49 | 0.538 | 0.101 |
| High (%, SD) | 34% | 0.48 | 36% | 0.49 | 0.750 | 0.050 | 32% | 0.47 | 36% | 0.49 | 0.618 | 0.081 |
| EQ-5D-5L (mean, SD) | 0.87 | 0.14 | 0.85 | 0.16 | 0.534 | 0.095 | 0.86 | 0.13 | 0.85 | 0.16 | 0.639 | 0.074 |
| EQ VAS (mean, SD) | 77.81 | 14.00 | 80.07 | 13.45 | 0.299 | 0.165 | 77.67 | 13.00 | 80.07 | 13.45 | 0.264 | 0.181 |
| SF12 PCS (mean, SD) | 48.98 | 7.80 | 46.92 | 10.22 | 0.122 | 0.227 | 48.04 | 8.18 | 46.92 | 10.22 | 0.439 | 0.121 |
| SF12 MCS (mean, SD) | 52.24 | 7.99 | 52.81 | 9.63 | 0.668 | 0.064 | 52.19 | 7.73 | 52.81 | 9.63 | 0.652 | 0.070 |
| BMI (mean, SD) | 26.53 | 4.36 | 27.2 | 4.38 | 0.329 | 0.153 | 26.87 | 4.25 | 27.2 | 4.38 | 0.630 | 0.078 |
| Smoking behaviour |  |  |  |  |  |  |  |  |  |  |  |  |
| Smoker (%, SD) | 17% | 0.37 | 18% | 0.39 | 0.798 | 0.040 | 15% | 0.36 | 18% | 0.39 | 0.597 | 0.085 |
| Former smoker (%, SD) | 54% | 0.50 | 44% | 0.50 | 0.195 | 0.204 | 57% | 0.50 | 44% | 0.50 | 0.110 | 0.261 |
| Non-smoker (%, SD) | 29% | 0.46 | 38% | 0.49 | 0.236 | 0.183 | 28% | 0.45 | 38% | 0.49 | 0.195 | 0.209 |
| Alcohol user (%, SD) | 64% | 0.48 | 65% | 0.48 | 0.858 | 0.028 | 63% | 0.48 | 65% | 0.48 | 0.788 | 0.044 |

| **Orthopaedics** | **Before PSM** | | | | | | **After PSM** | | | | | |
| --- | --- | --- | --- | --- | --- | --- | --- | --- | --- | --- | --- | --- |
|  | **PC+** | | **HBOC** | | **P-value** | **SMD** | **PC+** | | **HBOC** | | **P-value** | **SMD** |
| N | 339 | | 155 | |  |  | 294 | | 155 | |  |  |
| Age (mean, SD) | 57.35 | 13.47 | 60.66 | 13.02 | 0.011* | 0.250 | 58.88 | 12.60 | 60.66 | 13.02 | 0.160 | 0.139 |
| Gender (male) (%, SD) | 40% | 0.49 | 41% | 0.49 | 0.961 | 0.005 | 40% | 0.49 | 41% | 0.49 | 0.972 | 0.003 |
| Native country (Netherlands) (%, SD) | 96% | 0.21 | 95% | 0.22 | 0.719 | 0.034 | 95% | 0.21 | 95% | 0.22 | 0.853 | 0.018 |
| Educational level |  |  |  |  |  |  |  |  |  |  |  |  |
| Low (%, SD) | 19% | 0.39 | 28% | 0.45 | 0.021* | 0.218 | 21% | 0.41 | 28% | 0.45 | 0.095 | 0.163 |
| Medium (%, SD) | 50% | 0.50 | 54% | 0.50 | 0.340 | 0.093 | 47% | 0.50 | 54% | 0.50 | 0.126 | 0.152 |
| High (%, SD) | 32% | 0.47 | 18% | 0.39 | 0.001** | 0.322 | 33% | 0.47 | 18% | 0.39 | 0.001** | 0.339 |
| EQ-5D-5L (mean, SD) | 0.72 | 0.17 | 0.71 | 0.17 | 0.298 | 0.101 | 0.72 | 0.16 | 0.71 | 0.17 | 0.263 | 0.110 |
| EQ VAS (mean, SD) | 70.41 | 16.91 | 69.28 | 17.77 | 0.501 | 0.065 | 69.73 | 16.54 | 69.28 | 17.77 | 0.792 | 0.026 |
| SF12 PCS (mean, SD) | 41.64 | 8.77 | 40.29 | 9.77 | 0.126 | 0.146 | 40.91 | 8.56 | 40.29 | 9.77 | 0.489 | 0.067 |
| SF12 MCS (mean, SD) | 51.66 | 9.69 | 50.43 | 8.85 | 0.178 | 0.133 | 51.68 | 9.39 | 50.43 | 8.85 | 0.169 | 0.138 |
| BMI (mean, SD) | 27.06 | 4.64 | 27.34 | 4.84 | 0.533 | 0.060 | 27.32 | 4.74 | 27.34 | 4.84 | 0.959 | 0.005 |
| Smoking behaviour |  |  |  |  |  |  |  |  |  |  |  |  |
| Smoker (%, SD) | 15% | 0.36 | 15% | 0.36 | 0.953 | 0.006 | 15% | 0.36 | 15% | 0.36 | 0.971 | 0.004 |
| Former smoker (%, SD) | 40% | 0.49 | 46% | 0.50 | 0.235 | 0.115 | 40% | 0.49 | 46% | 0.50 | 0.248 | 0.114 |
| Non-smoker (%, SD) | 45% | 0.50 | 39% | 0.49 | 0.254 | 0.111 | 45% | 0.50 | 39% | 0.49 | 0.260 | 0.112 |
| Alcohol user (%, SD) | 59% | 0.49 | 55% | 0.50 | 0.427 | 0.077 | 60% | 0.49 | 55% | 0.50 | 0.411 | 0.082 |
| **Rheumatology** | **Before PSM** | | | | | | **After PSM** | | | | | |
|  | **PC+** | | **HBOC** | | **P-value** | **SMD** | **PC+** | | **HBOC** | | **P-value** | **SMD** |
| N | 120 | | 24 | |  |  | 48 | | 24 | |  |  |
| Age (mean, SD) | 55.08 | 13.32 | 58 | 14.15 | 0.334 | 0.212 | 55.5 | 11.66 | 58 | 14.15 | 0.428 | 0.193 |
| Gender (male) (%, SD) | 26% | 0.44 | 50% | 0.51 | 0.018* | 0.507 | 19% | 0.39 | 50% | 0.51 | 0.005** | 0.685 |
| Native country (Netherlands) (%, SD) | 98% | 0.13 | 100% | 0.00 | 0.528 | 0.183 | 96% | 0.20 | 100% | 0.00 | 0.317 | 0.292 |
| Educational level |  |  |  |  |  |  |  |  |  |  |  |  |
| Low (%, SD) | 26% | 0.44 | 21% | 0.41 | 0.609 | 0.117 | 31% | 0.47 | 21% | 0.41 | 0.359 | 0.235 |
| Medium (%, SD) | 50% | 0.50 | 42% | 0.50 | 0.459 | 0.166 | 52% | 0.50 | 42% | 0.50 | 0.412 | 0.207 |
| High (%, SD) | 24% | 0.43 | 38% | 0.49 | 0.178 | 0.288 | 17% | 0.38 | 38% | 0.49 | 0.051 | 0.474 |
| EQ-5D-5L (mean, SD) | 0.72 | 0.14 | 0.69 | 0.18 | 0.262 | 0.229 | 0.69 | 0.15 | 0.69 | 0.18 | 0.893 | 0.033 |
| EQ VAS (mean, SD) | 67.76 | 16.37 | 66.25 | 17.34 | 0.684 | 0.089 | 64.56 | 18.01 | 66.25 | 17.34 | 0.706 | 0.095 |
| SF12 PCS (mean, SD) | 41.50 | 8.60 | 40.68 | 8.64 | 0.668 | 0.096 | 39.04 | 9.37 | 40.68 | 8.64 | 0.475 | 0.182 |
| SF12 MCS (mean, SD) | 50.01 | 11.02 | 47.78 | 9.60 | 0.358 | 0.215 | 48.92 | 10.83 | 47.78 | 9.60 | 0.665 | 0.111 |
| BMI (mean, SD) | 26.55 | 4.68 | 27.04 | 7.11 | 0.671 | 0.081 | 26.71 | 4.38 | 27.04 | 7.11 | 0.811 | 0.055 |
| Smoking behaviour |  |  |  |  |  |  |  |  |  |  |  |  |
| Smoker (%, SD) | 22% | 0.41 | 29% | 0.46 | 0.428 | 0.171 | 23% | 0.42 | 29% | 0.46 | 0.570 | 0.140 |
| Former smoker (%, SD) | 49% | 0.50 | 33% | 0.48 | 0.158 | 0.322 | 42% | 0.50 | 33% | 0.48 | 0.501 | 0.170 |
| Non-smoker (%, SD) | 29% | 0.46 | 38% | 0.49 | 0.422 | 0.175 | 35% | 0.48 | 38% | 0.49 | 0.865 | 0.043 |
| Alcohol user (%, SD) | 57% | 0.50 | 58% | 0.50 | 0.881 | 0.033 | 48% | 0.50 | 58% | 0.50 | 0.412 | 0.207 |
| **Urology** | **Before PSM** | | | | | | **After PSM** | | | | | |
|  | **PC+** | | **HBOC** | | **P-value** | **SMD** | **PC+** | | **HBOC** | | **P-value** | **SMD** |
| N | 24 | | 37 | |  |  | 24 | | 22 | |  |  |
| Age (mean, SD) | 56.25 | 19.09 | 55 | 15.61 | 0.781 | 0.072 | 56.25 | 19.09 | 56.55 | 17.03 | 0.956 | 0.016 |
| Gender (male) (%, SD) | 79% | 0.41 | 62% | 0.49 | 0.167 | 0.374 | 79% | 0.41 | 0.64 | 0.49 | 0.252 | 0.341 |
| Native country (Netherlands) (%, SD) | 100% | 0.00 | 97% | 0.16 | 0.425 | 0.232 | 100% | 0.00 | 95% | 0.21 | 0.301 | 0.302 |
| Educational level |  |  |  |  |  |  |  |  |  |  |  |  |
| Low (%, SD) | 29% | 0.46 | 16% | 0.37 | 0.235 | 0.307 | 29% | 0.46 | 18% | 0.39 | 0.394 | 0.255 |
| Medium (%, SD) | 50% | 0.51 | 43% | 0.50 | 0.612 | 0.133 | 50% | 0.51 | 45% | 0.51 | 0.764 | 0.089 |
| High (%, SD) | 21% | 0.41 | 41% | 0.50 | 0.113 | 0.430 | 21% | 0.41 | 36% | 0.49 | 0.252 | 0.341 |
| EQ-5D-5L (mean, SD) | 0.82 | 0.20 | 0.85 | 0.14 | 0.568 | 0.145 | 0.82 | 0.20 | 0.86 | 0.12 | 0.447 | 0.229 |
| EQ VAS (mean, SD) | 72.79 | 19.45 | 75.68 | 11.92 | 0.475 | 0.179 | 72.79 | 19.45 | 73.91 | 9.55 | 0.809 | 0.073 |
| SF12 PCS (mean, SD) | 49.14 | 6.96 | 48.72 | 8.40 | 0.840 | 0.054 | 49.14 | 6.96 | 49.31 | 6.84 | 0.932 | 0.025 |
| SF12 MCS (mean, SD) | 49.90 | 10.29 | 49.2 | 8.25 | 0.768 | 0.076 | 49.9 | 10.29 | 49.11 | 7.49 | 0.770 | 0.087 |
| BMI (mean, SD) | 27.47 | 5.26 | 25.04 | 3.97 | 0.045* | 0.520 | 27.47 | 5.26 | 25.24 | 3.38 | 0.097 | 0.505 |
| Smoking behaviour |  |  |  |  |  |  |  |  |  |  |  |  |
| Smoker (%, SD) | 12% | 0.34 | 14% | 0.35 | 0.911 | 0.030 | 12% | 0.34 | 18% | 0.39 | 0.602 | 0.155 |
| Former smoker (%, SD) | 46% | 0.51 | 46% | 0.51 | 0.993 | 0.002 | 46% | 0.51 | 45% | 0.51 | 0.980 | 0.007 |
| Non-smoker (%, SD) | 42% | 0.50 | 41% | 0.50 | 0.932 | 0.022 | 42% | 0.50 | 36% | 0.49 | 0.720 | 0.106 |
| Alcohol user (%, SD) | 50% | 0.51 | 62% | 0.49 | 0.356 | 0.243 | 50% | 0.51 | 59% | 0.50 | 0.547 | 0.179 |

*PC+ = Primary Care Plus; HBOC = Hospital Based Outpatient Care; PSM = Propensity score matching; SMD = standardized mean differences; SD = standard deviation*

** P < 0.05; ** P < 0.01; *** P < 0.001*
