# Supplementary material for: Patients' perspectives on a new delivery model in primary care: A propensity score matched analysis of patient‐reported outcomes in a Dutch cohort study
Source: J Eval Clin Pract. 2020 Jun 17;27(2):344–55. doi: 10.1111/jep.13426 (PMC7983912; doi:10.1111/jep.13426)
Supplement: Supplementary file 3 — TABLE S3. Health‐Related Quality of Life Outcomes Before and After Propensity Score Matching per Medical Specialty. [file JEP-27-344-s002.docx]

**Table S3** Health-Related Quality of Life Outcomes Before and After Propensity Score Matching per Medical Specialty

|  | **Dermatology** | | **Gynaecology** | | **Otolaryngology** | | **Internal medicine** | | **Neurology** | | **Ophthalmology** | | **Orthopaedics** | | **Rheumatology** | | **Urology** | |
| --- | --- | --- | --- | --- | --- | --- | --- | --- | --- | --- | --- | --- | --- | --- | --- | --- | --- | --- |
|  | Before  PSM | After  PSM | Before  PSM | After  PSM | Before  PSM | After  PSM | Before  PSM | After  PSM | Before  PSM | After  PSM | Before  PSM | After  PSM | Before  PSM | After  PSM | Before  PSM | After  PSM | Before  PSM | After  PSM |
| **EQ-5D-5L** |  |  |  |  |  |  |  |  |  |  |  |  |  |  |  |  |  |  |
| Time T1 x study group ^†^ | - | - | - | - | - | - | - | - | PC+  * | PC+  * | - | - | - | - | - | - | - | - |
| Time T2 x study group ^†^ | - | - | - | - | - | - | - | - | PC+  * | - | PC+  * | - | - | - | - | - | - | - |
| **EQ-VAS** |  |  |  |  |  |  |  |  |  |  |  |  |  |  |  |  |  |  |
| Time T1 x study group ^†^ | - | - | - | - | - | - | - | - | PC+  * | PC+  * | - | - | - | - | - | - | - | - |
| Time T2 x study group ^†^ | - | - | - | - | - | - | - | - | PC+  * | PC+  * | - | - | - | - | - | - | - | - |
| **SF-12 PCS** |  |  |  |  |  |  |  |  |  |  |  |  |  |  |  |  |  |  |
| Time T2 x study group ^†^ | HBOC  * | HBOC  * | - | - | - | - | - | - | - | - | - | - | - | - | - | - | - | - |
| **SF-12 MCS** |  |  |  |  |  |  |  |  |  |  |  |  |  |  |  |  |  |  |
| Time T2 x study group ^†^ | - | - | - | - | - | PC+  * | - | PC+  * | - | - | - | - | - | - | - | - | - | - |
| **PGIC ^‡^** |  |  |  |  |  |  |  |  |  |  |  |  |  |  |  |  |  |  |
| Time T2 x study group ^†^ | - | - | - | - | PC+  * | PC+  * | - | - | - | - | - | - | - | - | - | - | - | - |

*PC+ = Primary Care Plus; HBOC = Hospital Based Outpatient Care; PSM = Propensity score matching;*

*In table: PC+ = significant higher scores in the PC+ group; HBOC = significant higher scores in the HBOC group*

*^†^ Group was coded as 1 = HBOC group and 0 = PC+ group; ^‡^ PGIC was measured at T1 and T2, not at baseline*

** P < 0.05*
