# Supplementary material for: Patients' perspectives on a new delivery model in primary care: A propensity score matched analysis of patient‐reported outcomes in a Dutch cohort study
Source: J Eval Clin Pract. 2020 Jun 17;27(2):344–55. doi: 10.1111/jep.13426 (PMC7983912; doi:10.1111/jep.13426)
Supplement: Supplementary file 4 — TABLE S4. Comparison of Patient Experienced Quality of Care Outcomes Before and After Propensity Score Matching per Medical Specialty. [file JEP-27-344-s004.docx]

**Table S4** Comparison of Patient Experienced Quality of Care Outcomes Before and After Propensity Score Matching per Medical Specialty

|  | **Dermatology** | | **Gynaecology** | | **Otolaryngology** | | **Internal medicine** | | **Neurology** | | **Ophthalmology** | | **Orthopaedics** | | **Rheumatology** | | **Urology** | |
| --- | --- | --- | --- | --- | --- | --- | --- | --- | --- | --- | --- | --- | --- | --- | --- | --- | --- | --- |
|  | Before  PSM | After  PSM | Before  PSM | After  PSM | Before  PSM | After  PSM | Before  PSM | After  PSM | Before  PSM | After  PSM | Before  PSM | After  PSM | Before  PSM | After  PSM | Before  PSM | After  PSM | Before  PSM | After  PSM |
| **Timeliness (1)** |  |  |  |  |  |  |  |  |  |  |  |  |  |  |  |  |  |  |
| Waiting time for  appointment | - | - | - | - | - | - | - | - | - | - | - | - | - | - | - | - | PC+  * | - |
| Waiting time in waiting  room <30 minutes | PC+ ** | PC+ ** | - | - | - | - | - | - | - | - | - | - | - | - | - | - | - | - |
| **Treatment by the medical specialist** | | | | | | | | | | | | | | | | | | |
| Complaint was taken  seriously | - | - | - | - | - | - | - | - | - | - | - | - | - | - | - | - | - | - |
| Specialist listened  carefully | - | - | - | - | - | - | - | - | - | - | - | - | - | - | - | - | - | - |
| Specialist took enough  time | - | - | - | - | - | - | - | - | - | - | - | - | - | - | - | - | - | - |
| Treated with respect | - | - | - | - | - | - | - | - | - | - | - | - | - | - | - | - | - | - |
| Competence of the  specialist | - | - | - | - | - | - | - | - | - | - | - | - | - | - | - | - | - | - |
| Overall help of the  specialist | - | - | - | - | - | - | - | - | - | - | - | - | - | - | - | - | - | - |
| **Information provision and communication by the medical specialist** | | | | | | | | | | | | | | | | | | |
| Information about  different treatment  options | - | - | - | - | - | - | - | - | - | - | - | - | - | - | - | - | - | - |
| Understandable  explanation | - | - | - | - | - | - | - | - | - | - | PC+  * | - | - | - | - | - | - | - |
| Opportunity to ask  questions | - | PC+  * | - | - | - | - | - | - | PC+  * | PC+  * | - | - | - | - | - | - | - | - |
| Shared decision making | - | - | - | - | - | - | - | - | - | - | - | - | - | - | - | - | - | - |
| **Communication and collaboration between the GP and medical specialist** | | | | | | | | | | | | | | | | | | |
| Matching  recommendations  between GP and  specialist | - | - | - | - | - | - | - | - | - | - | - | - | - | - | - | - | - | - |
| Awareness of the medical  specialist about the  complaint | - | - | - | - | - | - | - | - | - | - | - | - | - | - | - | - | - | - |
| Collaboration and  alignment between GP  and specialist | - | - | - | - | - | - | - | - | - | - | - | - | - | - | - | - | - | - |
| **Overall assessment of quality of care (1)** | | | | | | | | | | | | | | | | | | |
| Recommend medical  specialist to  family/friends | - | - | - | - | - | - | - | - | - | - | - | - | - | - | - | - | - | - |
| Recommend PC+/HBOC  to family/friends | - | - | - | - | - | - | - | - | - | - | - | - | - | - | - | - | - | - |
| **Timeliness (2)** | | | | | | | | | | | | | | | | | | |
| Travel time (in minutes) ^†^ | HBOC ** | HBOC ** | - | - | HBOC ** | HBOC  * | - | - | - | - | - | - | HBOC  ** | HBOC  ** | - | - | - | - |
| **Overall assessment of quality of care (2)** | | | | | | | | | | | | | | | | | | |
| Grade specialist (0-10) | - | - | - | - | - | - | - | - | - | - | - | - | - | - | - | - | - | - |
| Grade PC+/HBOC (0-10) | PC+ ** | PC+ * | - | - | - | - | - | - | - | - | - | - | - | - | - | - | - | - |

*PC+ = Primary Care Plus; HBOC = Hospital Based Outpatient Care; PSM = Propensity score matching;*

*In table: PC+ = significant higher scores in the PC+ group; HBOC = significant higher scores in the HBOC group*

^†^ *A significant higher score on travel time means a longer travel time in minutes and is in this case a unfavourable result*

** P < 0.01; ** P < 0.00*
