# Supplementary material for: Patients' perspectives on a new delivery model in primary care: A propensity score matched analysis of patient‐reported outcomes in a Dutch cohort study
Source: J Eval Clin Pract. 2020 Jun 17;27(2):344–55. doi: 10.1111/jep.13426 (PMC7983912; doi:10.1111/jep.13426)
Supplement: Supplementary file 5 — TABLE S5. Baseline Characteristics Before and After Propensity Score Matching. [file JEP-27-344-s001.docx]

**Table S5** Baseline Characteristics Before and After Propensity Score Matching

|  | **Before PSM** | | | | | | **After PSM** | | | | | |
| --- | --- | --- | --- | --- | --- | --- | --- | --- | --- | --- | --- | --- |
|  | **PC+** | | **HBOC** | | **P-value** | **SMD** | **PC+** | | **HBOC** | | **P-value** | **SMD** |
| **N** | **1763** | | **643** | |  |  | **608** | | **608** | |  |  |
| Age (mean, SD) | 55.95 | 15.68 | 57.63 | 15.23 | 0.019* | 0.109 | 57.25 | 15.10 | 57.81 | 15.18 | 0.516 | 0.037 |
| Gender (male) (%, SD) | 39% | 0.49 | 41% | 0.49 | 0.557 | 0.027 | 43% | 0.50 | 41% | 0.49 | 0.450 | 0.043 |
| Native country (Netherlands) (%, SD) | 97% | 0.18 | 96% | 0.20 | 0.393 | 0.038 | 95% | 0.21 | 96% | 0.20 | 0.679 | 0.024 |
| Educational level |  |  |  |  |  |  |  |  |  |  |  |  |
| Low (%, SD) | 19% | 0.39 | 22% | 0.42 | 0.063 | 0.084 | 21% | 0.41 | 22% | 0.42 | 0.677 | 0.024 |
| Medium (%, SD) | 47% | 0.50 | 46% | 0.50 | 0.593 | 0.025 | 46% | 0.50 | 46% | 0.50 | 0.954 | 0.003 |
| High (%, SD) | 34% | 0.48 | 32% | 0.47 | 0.317 | 0.046 | 33% | 0.47 | 32% | 0.47 | 0.759 | 0.018 |
| EQ-5D-5L (mean, SD) | 0.81 | 0.17 | 0.79 | 0.17 | 0.005^**^ | 0.129 | 0.79 | 0.17 | 0.79 | 0.17 | 0.866 | 0.010 |
| EQ VAS (mean, SD) | 75.53 | 16.32 | 73.08 | 16.31 | 0.001^**^ | 0.150 | 73.17 | 16.76 | 73.03 | 16.38 | 0.883 | 0.008 |
| SF12 PCS (mean, SD) | 47.44 | 9.33 | 45.39 | 10.04 | ≤0.001^***^ | 0.211 | 45.73 | 9.78 | 45.34 | 10.06 | 0.489 | 0.040 |
| SF12 MCS (mean, SD) | 51.22 | 9.35 | 50.11 | 9.34 | 0.010^**^ | 0.119 | 49.97 | 9.59 | 50.23 | 9.39 | 0.628 | 0.028 |
| BMI (mean, SD) | 26.20 | 4.60 | 26.50 | 4.92 | 0.170 | 0.062 | 26.54 | 4.65 | 26.53 | 4.82 | 0.984 | 0.001 |
| Smoking behaviour |  |  |  |  |  |  |  |  |  |  |  |  |
| Smoker (%, SD) | 16% | 0.36 | 17% | 0.38 | 0.443 | 0.035 | 16% | 0.37 | 17% | 0.38 | 0.758 | 0.018 |
| Former smoker (%, SD) | 42% | 0.49 | 42% | 0.49 | 0.771 | 0.013 | 45% | 0.50 | 42% | 0.49 | 0.326 | 0.056 |
| Non-smoker (%, SD) | 42% | 0.49 | 41% | 0.49 | 0.781 | 0.013 | 39% | 0.49 | 41% | 0.49 | 0.447 | 0.044 |
| Alcohol user (%, SD) | 62% | 0.48 | 60% | 0.49 | 0.198 | 0.059 | 61% | 0.49 | 59% | 0.49 | 0.482 | 0.040 |
| Medical specialty referred to |  |  |  |  |  |  |  |  |  |  |  |  |
| Dermatology (%, SD) | 32% | 0.47 | 16% | 0.37 | ≤0.001^***^ | 0.371 | 17% | 0.38 | 17% | 0.38 | 0.940 | 0.004 |
| Gynaecology (%, SD) | 5% | 0.22 | 7% | 0.25 | 0.260 | 0.051 | 8% | 0.27 | 7% | 0.25 | 0.511 | 0.038 |
| Internal medicine (%, SD) | 2% | 0.15 | 9% | 0.29 | <0.001^***^ | 0.307 | 7% | 0.25 | 7% | 0.26 | 0.733 | 0.020 |
| Otolaryngology (%, SD) | 17% | 0.37 | 13% | 0.34 | 0.046 | 0.094 | 13% | 0.34 | 14% | 0.35 | 0.740 | 0.019 |
| Neurology (%, SD) | 7% | 0.26 | 12% | 0.33 | ≤0.001^***^ | 0.159 | 13% | 0.33 | 12% | 0.33 | 0.931 | 0.005 |
| Ophthalmology (%, SD) | 9% | 0.28 | 9% | 0.28 | 0.821 | 0.010 | 9% | 0.28 | 9% | 0.29 | 0.762 | 0.017 |
| Orthopaedics (%, SD) | 19% | 0.39 | 24% | 0.43 | 0.009^**^ | 0.119 | 26% | 0.44 | 25% | 0.44 | 0.896 | 0.008 |
| Rheumatology (%, SD) | 7% | 0.25 | 4% | 0.19 | 0.005^**^ | 0.138 | 4% | 0.19 | 4% | 0.19 | 1.000 | 0.001 |
| Urology (%, SD) | 1% | 0.12 | 6% | 0.23 | ≤0.001^***^ | 0.239 | 4% | 0.19 | 4% | 0.19 | 1.000 | 0.001 |
| *PC+ = Primary Care Plus; HBOC = Hospital Based Outpatient Care; PSM = Propensity score matching; SMD = standardized mean differences; SD = standard deviation* | | | | | | | | | | | | |

** P < 0.05; ** P < 0.01; *** P < 0.000*
