# Supplementary material for: Patients' perspectives on a new delivery model in primary care: A propensity score matched analysis of patient‐reported outcomes in a Dutch cohort study
Source: J Eval Clin Pract. 2020 Jun 17;27(2):344–55. doi: 10.1111/jep.13426 (PMC7983912; doi:10.1111/jep.13426)
Supplement: Supplementary file 6 — TABLE S6. Health‐Related Quality of Life Outcomes Before and After Propensity Score Matching. [file JEP-27-344-s008.docx]

**Table S6** Health-Related Quality of Life Outcomes Before and After Propensity Score Matching

|  | **Before PSM** | | | **After PSM** | | |
| --- | --- | --- | --- | --- | --- | --- |
| **EQ-5D-5L** | **Estimate** | **SE** | **95%CI** | **Estimate** | **SE** | **95%CI** |
| Intercept | 0.82^***^ | 0.00 | 0.81 - 0.82 | 0.79^***^ | 0.01 | 0.78 – 0.81 |
| Study group ^†^ | -0.02^**^ | 0.01 | -0.04 - -0.01 | -0.00 | 0.01 | -0.02- 0.02 |
| Time T1 | 0.01^***^ | 0.00 | 0.01 - 0.02 | 0.01 | 0.01 | 0.00 – 0.03 |
| Time T2 | 0.02^***^ | 0.00 | 0.01 - 0.02 | 0.01^*^ | 0.00 | 0.00 – 0.02 |
| Time T1 x study group | -0.01* | 0.01 | -0.02 - 0.00 | -0.01 | 0.01 | -0.03 – 0.01 |
| Time T2 x study group | -0.01^*^ | 0.01 | -0.03 - -0.00 | -0.01 | 0.01 | -0.02 – 0.00 |
| **EQ-VAS** | **Estimate** | **SE** | **95%CI** | **Estimate** | **SE** | **95%CI** |
| Intercept | 75.53^***^ | 0.39 | 74.77 – 76.29 | 73.17^***^ | 0.67 | 71.85 – 74.49 |
| Study group ^†^ | -2.45^**^ | 0.75 | -3.92 – -0.97 | -1.14 | 0.95 | -2.00 – 1.72 |
| Time T1 | 0.92^**^ | 0.31 | 0.32 – 1.53 | 0.84 | 0.48 | -0.11 – 1.79 |
| Time T2 | 1.10 | 0.64 | -0.16 – 2.35 | 1.29^*^ | 0.60 | 0.10 – 2.47 |
| Time T1 x study group | -0.41 | 0.60 | -1.58 – 0.77 | -0.39 | 0.68 | -1.73 – 0.95 |
| Time T2 x study group | -0.16 | 1.25 | -2.60 – 2.28 | -0.61 | 0.86 | -2.29 – 1.06 |
| **SF-12 PCS** | **Estimate** | **SE** | **95%CI** | **Estimate** | **SE** | **95%CI** |
| Intercept | 47.44^***^ | 0.23 | 46.99 – 47.88 | 45.73^***^ | 0.40 | 44.94 – 46.52 |
| Study group ^†^ | -2.05^***^ | 0.44 | -2.91 – -1.19 | -0.40 | 0.57 | -1.51 – 0.72 |
| Time T2 | 0.57^***^ | 0.17 | 0.24 – 0.90 | 1.31^***^ | 0.29 | 0.74 – 1.87 |
| Time T2 x study group | 0.11 | 0.33 | -0.53 – 0.75 | -0.63 | 0.41 | -1.44 – 0.17 |
| **SF-12 MCS** | **Estimate** | **SE** | **95%CI** | **Estimate** | **SE** | **95%CI** |
| Intercept | 51.22^***^ | 0.22 | 50.78 – 51.66 | 49.97^***^ | 0.38 | 49.21 – 51.72 |
| Study group ^†^ | -1.11^**^ | 0.43 | -1.95 – -0.27 | 0.26 | 0.54 | -0.80 – 1.33 |
| Time T2 | 0.03 | 0.20 | -0.36 – 0.42 | 0.22 | 0.35 | -0.37 – 1.00 |
| Time T2 x study group | -0.32 | 0.39 | -1.08 – 0.44 | -0.74 | 0.50 | -1.71 – 0.23 |
| **PGIC** *^‡^* | **Estimate** | **SE** | **95%CI** | **Estimate** | **SE** | **95%CI** |
| Intercept | 4.57^***^ | 0.73 | 3.14 – 6.01 | 3.44^***^ | 0.04 | 3.36 – 3.51 |
| Study group ^†^ | -1.10 | 1.41 | -3.88 – 1.67 | 0.05 | 0.05 | -0.05 – 0.16 |
| Time T2 | -1.58^*^ | 0.73 | -3.02 - -0.15 | -0.35^***^ | 0.05 | -0.46 - -0.24 |
| Time T2 x study group | 1.11 | 1.42 | -1.67 – 3.89 | -0.15 | 0.08 | -0.30 – 0.00 |

*PSM = Propensity score matching^;^ SE = Standard Error; CI = Confidence Interval*

^†^ *Group was coded as 1 = Hospital Based Outpatient Care (HBOC) group and 0 = Primary Care Plus (PC+) group; ^‡^ PGIC was measured at T1 and T2, not at baseline*

** P < 0.05; ** P < 0.01; *** P < 0.00*
