# Supplementary material for: Patients' perspectives on a new delivery model in primary care: A propensity score matched analysis of patient‐reported outcomes in a Dutch cohort study
Source: J Eval Clin Pract. 2020 Jun 17;27(2):344–55. doi: 10.1111/jep.13426 (PMC7983912; doi:10.1111/jep.13426)
Supplement: Supplementary file 7 — TABLE S7. Comparison of Patient Experienced Quality of Care Outcomes Before and After Propensity Score Matching. [file JEP-27-344-s006.docx]

**Table S7** Comparison of Patient Experienced Quality of Care Outcomes Before and After Propensity Score Matching

|  | **Before PSM** | | | **After PSM** | | |
| --- | --- | --- | --- | --- | --- | --- |
|  | **PC+** | **HBOC** |  | **PC+** | **HBOC** |  |
| **N** | **1741** | **624** |  | **600** | **589** |  |
| **Quality of care domains** | **Satisfied/Yes n (%)** | **Satisfied/Yes n (%)** | **P-value** | **Satisfied/Yes n (%)** | **Satisfied/Yes n (%)** | **P-value** |
| **Timeliness (1)** |  |  |  |  |  |  |
| Waiting time for appointment | 89.2(1527) | 86.0(533) | 0.032 | 90.2(535) | 86.5(507) | 0.047 |
| Waiting time in waiting room <30 minutes | 93.5 (1605) | 88.5 (546) | ≤0.001^**^ | 91.9(545) | 88(514) | 0.026 |
| **Treatment by the medical specialist** | | |  |  |  |  |
| Complaint was taken seriously | 97.5(1672) | 97.4(601) | 0.845 | 98.1(583) | 97.3(568) | 0.309 |
| Specialist listened carefully | 97.3(1667) | 97.4(601) | 0.845 | 98.3(584) | 97.3(568) | 0.217 |
| Specialist took enough time | 98.0(1679) | 98.7(608) | 0.240 | 98.5(585) | 98.6(575) | 0.837 |
| Treated with respect | 98.8(1692) | 98.5(607) | 0.574 | 99.2(589) | 98.5(574) | 0.267 |
| Competence of the specialist | 98.4(1673) | 98.0(601) | 0.543 | 99.2(586) | 98.1(569) | 0.122 |
| Overall help of the specialist | 94.2(1612) | 93.5(575) | 0.553 | 94.8(562) | 93.3(543) | 0.286 |
| **Information provision and communication by the medical specialist** | | | |  |  |  |
| Information about different treatment options | 92.6(1581) | 90.7(555) | 0.140 | 92.6(550) | 90.3(523) | 0.165 |
| Understandable explanation | 97.1(1663) | 96.1(592) | 0.236 | 97.0(576) | 95.9(559) | 0.315 |
| Opportunity to ask questions | 97.4(1666) | 96.4(594) | 0.231 | 97.6(579) | 96.2(561) | 0.160 |
| Shared decision making | 88.4(892) | 87.3(338) | 0.582 | 89.5(315) | 86.7(320) | 0.252 |
| **Communication and collaboration between the GP and medical specialist** | | | |  |  |  |
| Matching recommendations between GP and specialist | 80.3(1357) | 82.1(501) | 0.324 | 80.6(473) | 82.0(473) | 0.165 |
| Awareness of the medical specialist about the complaint | 89.4(1519) | 89.1(547) | 0.827 | 89.3(527) | 89.5(520) | 0.921 |
| Collaboration and alignment between GP and specialist | 85.8(1366) | 81.6(482) | 0.016 | 85.6(476) | 81.5(455) | 0.067 |
| **Overall assessment of quality of care (1)** | | |  |  |  |  |
| Recommend medical specialist to family/friends | 93.7(1598) | 92.5(568) | 0.298 | 94.9(563) | 92.1(535) | 0.047 |
| Recommend PC+/HBOC to family/friends | 95.4(1625) | 93.8(577) | 0.119 | 95.8(568) | 93.8(546) | 0.128 |
|  | **Mean (SD)** | **Mean (SD)** |  | **Mean (SD)** | **Mean (SD)** |  |
| **Timeliness (2)** | | | |  |  |  |
| Travel time (in minutes) ^†^ | 15.6 (9.34) | 19.2 (12.18) | ≤0.001^**^ | 16.3 (9.29) | 16.8 (11.18) | 0.212 |
| **Overall assessment of quality of care (2)** | | |  |  |  |  |
| Grade specialist (0-10) | 8.5 (1.15) | 8.4 (1.22) | 0.007^*^ | 8.5 (1.20) | 8.4 (1.14) | 0.048 |
| Grade PC+/HBOC (0-10) | 8.5 (1.08) | 8.3 (1.11) | ≤0.001^**^ | 8.5 (1.06) | 8.4 (1.11) | 0.014 |

*PC+ = Primary Care Plus; HBOC = Hospital Based Outpatient Care; PSM = Propensity score matching;*

*SD = Standard Deviation*

*^†^ A significant higher score on travel time means a longer travel time in minutes and is in this case a unfavourable result*

** P < 0.01; ** P < 0.00*
