# Supplementary figures and images for: Patients' perspectives on a new delivery model in primary care: A propensity score matched analysis of patient‐reported outcomes in a Dutch cohort study
Source: J Eval Clin Pract. 2020 Jun 17;27(2):344–55. doi: 10.1111/jep.13426 (PMC7983912; doi:10.1111/jep.13426)

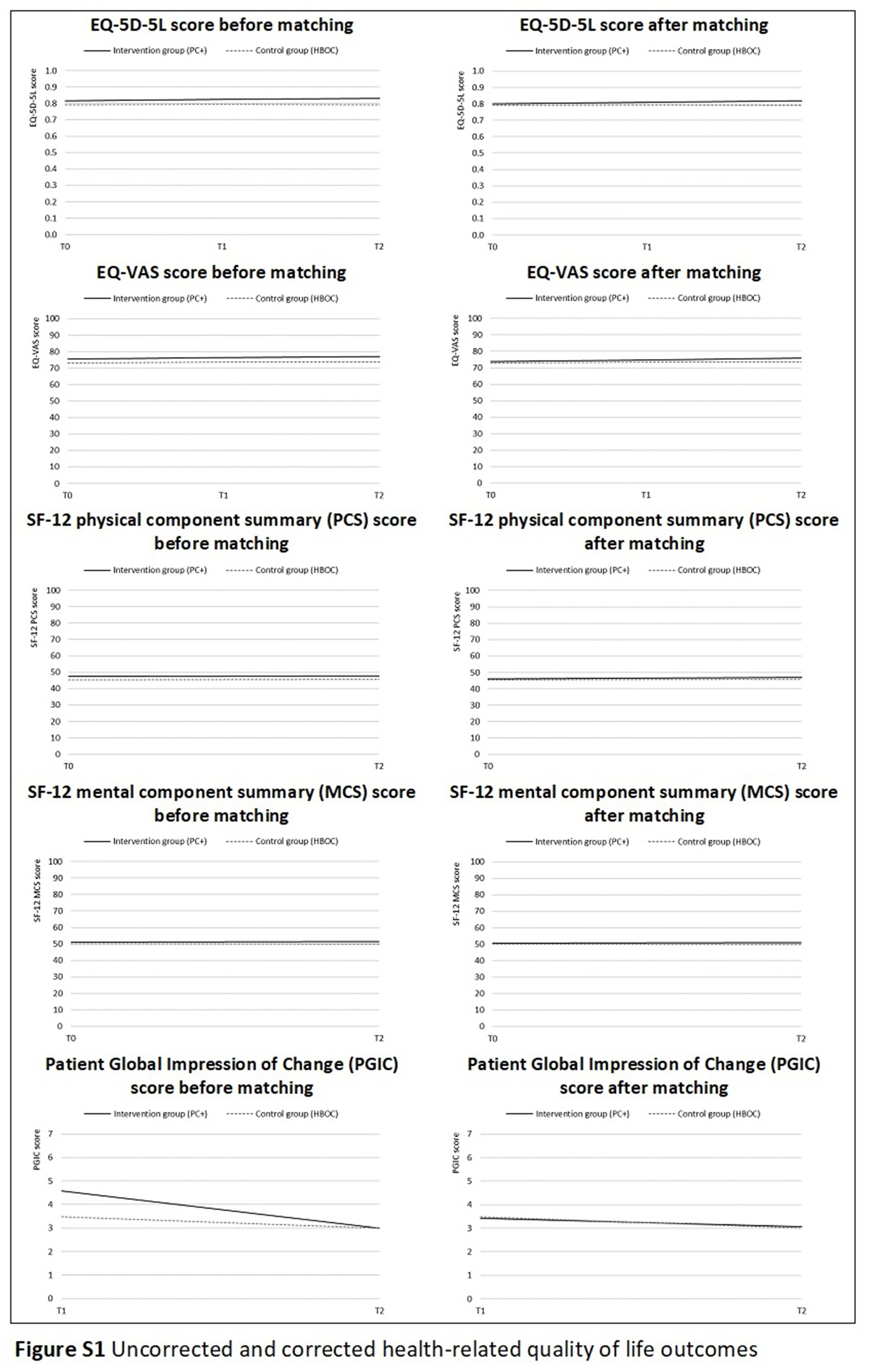

Supplement: Supplementary file 8 — FIGURE S1. Uncorrected and corrected health‐related quality of life outcomes. [file JEP-27-344-s007.tif]

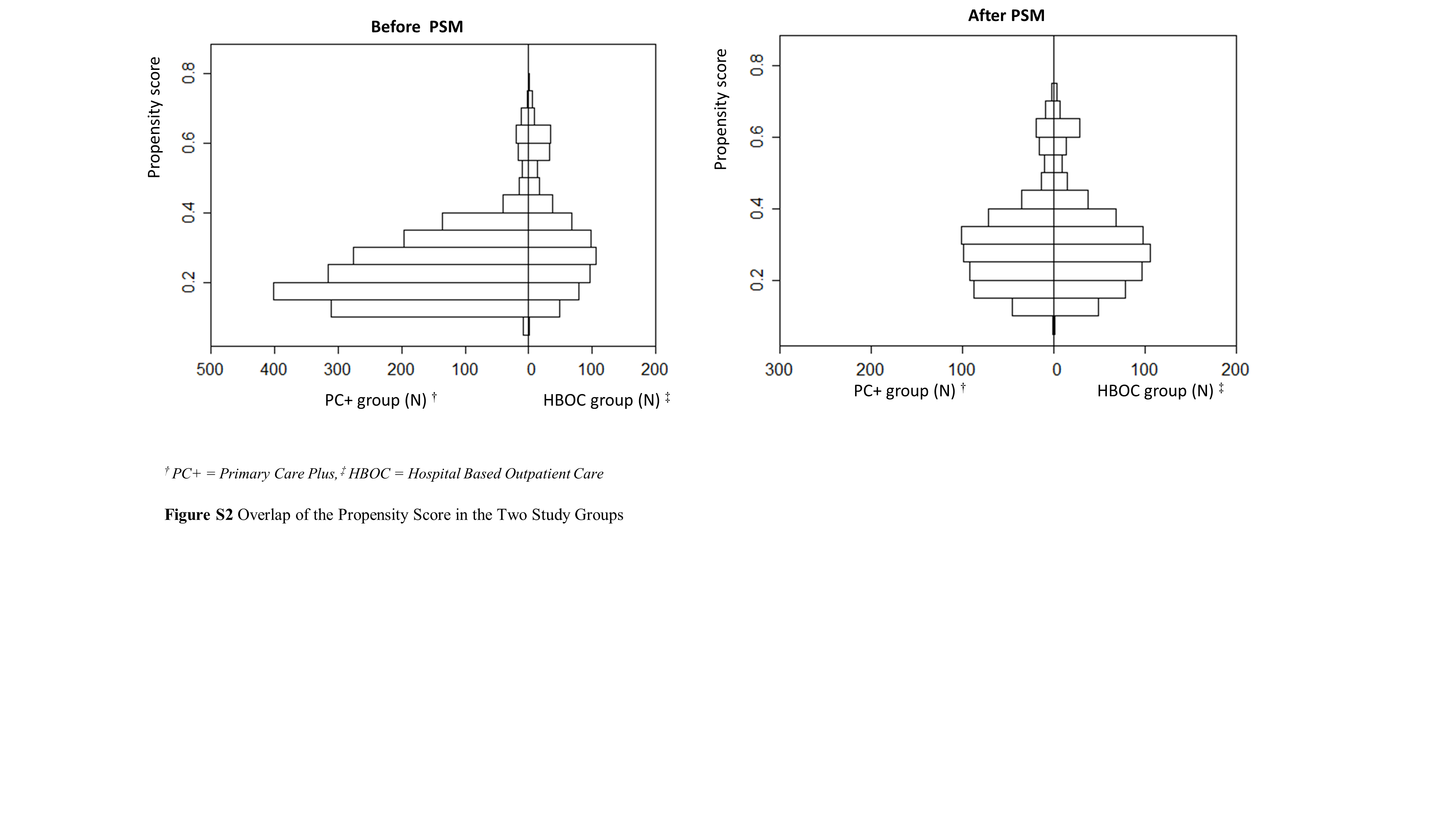

Supplement: Supplementary file 9 — FIGURE S2. Overlap of the Propensity Score in the Two Study Groups. [file JEP-27-344-s003.tif]
